# Redefining the Subsurface Biosphere: Characterization of Fungi Isolated From Energy-Limited Marine Deep Subsurface Sediment

**DOI:** 10.3389/ffunb.2021.727543

**Published:** 2021-09-24

**Authors:** Brandi Kiel Reese, Morgan S. Sobol, Marshall Wayne Bowles, Kai-Uwe Hinrichs

**Affiliations:** ^1^Dauphin Island Sea Lab, Dauphin Island, AL, United States; ^2^School of Marine and Environmental Sciences, University of South Alabama, Mobile, AL, United States; ^3^Hanse-Wissenschaftskolleg Institute for Advanced Study, Delmenhorst, Germany; ^4^Institute for Biological Interfaces 5, Karlsruhe Institute of Technology, Eggenstein-Leopoldshafen, Germany; ^5^Department of Life Sciences, Texas A&M University, Corpus Christi, TX, United States; ^6^Louisiana Universities Marine Consortium, Cocodrie, LA, United States; ^7^MARUM-Center for Marine Environmental Sciences, University of Bremen, Bremen, Germany

**Keywords:** fungi, marine sediment, subsurface, IODP (Integrated Ocean Drilling Program) Expedition 329, oligotrophic

## Abstract

The characterization of metabolically active fungal isolates within the deep marine subsurface will alter current ecosystem models and living biomass estimates that are limited to bacterial and archaeal populations. Although marine fungi have been studied for over fifty years, a detailed description of fungal populations within the deep subsurface is lacking. Fungi possess metabolic pathways capable of utilizing previously considered non-bioavailable energy reserves. Therefore, metabolically active fungi would occupy a unique niche within subsurface ecosystems, with the potential to provide an organic carbon source for heterotrophic prokaryotic populations from the transformation of non-bioavailable energy into substrates, as well as from the fungal necromass itself. These organic carbon sources are not currently being considered in subsurface energy budgets. Sediments from South Pacific Gyre subsurface, one of the most energy-limited environments on Earth, were collected during the Integrated Ocean Drilling Program Expedition 329. Anoxic and oxic sediment slurry enrichments using fresh sediment were used to isolate multiple fungal strains in media types that varied in organic carbon substrates and concentration. Metabolically active and dormant fungal populations were also determined from nucleic acids extracted from *in situ* cryopreserved South Pacific Gyre sediments. For further characterization of physical growth parameters, two isolates were chosen based on their representation of the whole South Pacific Gyre fungal community. Results from this study show that fungi have adapted to be metabolically active and key community members in South Pacific Gyre sediments and potentially within global biogeochemical cycles.

## Introduction

Fungi can utilize a range of mechanisms to cope within extreme habitats and in those with limited sources of organic carbon and nutrients. In general, the specific fungal life strategies have been classified into ruderal, stress selected, and combative (Boddy and Hiscox, 2016). Most relevant to deep subsurface conditions would be stress selected and combative fungal phylotypes. Osmotic stress selected fungal spores can survive at a water activity just above 0.55, when most organisms are desiccated at a water activity of around 0.996 (Pugh et al., 1983). Fungi classified as combative also gain organic nutrients from recalcitrant sources by utilizing a range of enzymes to breakdown complex organic molecules such as cellulose, lignin, and other recalcitrant carbon compounds and defend themselves using a variety of processes (i.e., antibiotics, mycoparasitism, and contact inhibition) (Kirk and Farrell, 1987; Ruiz-Dueñas and Martínez, 2009). Many marine fungi also use nitrate as their sole nitrogen source, converting it to ammonium by the enzymes nitrate reductase and nitrite reductase (Griffin, 1996; Cathrine and Raghukumar, 2009; Mouton et al., 2012; Stief et al., 2014). These fungal survival strategies of stress selection, combativeness, and sporulation warrant the investigation of metabolically active fungi in the deep subsurface.

It is estimated that Earth's biosphere contains ~550 gigatons of biomass in the form of carbon, of which ~1.8% of this biomass is made up of prokaryotes in the marine subsurface (Bar-On et al., 2018). Previous estimates failed to consider the presence of fungal eukaryotes (Kallmeyer et al., 2012; Orcutt et al., 2013), therefore the amount of fungal biomass has remained largely uncertain in marine and subsurface environments and should be interpreted with caution (Bar-On et al., 2018). Recent studies have provided the initial descriptions of fungal populations in shallow sediments (<37 m) on the continental shelf, near hydrothermal vents where organic matter is abundant compared to the vast abyssal plains, and several deep-sea marine sediments (Raghukumar et al., 2004; Damare et al., 2006; Singh et al., 2010; Thaler et al., 2012) and marine subsurface locations, including depths as far as 1,884 m below seafloor (mbsf) along a continental slope off the coast of New Zealand (Edgcomb et al., 2011; Orsi et al., 2013; Rédou et al., 2015; Liu et al., 2017). However, South Pacific Gyre (SPG) sediments represent an isolated, low-carbon end member in the marine carbon cycle and an ideal location to understand the tolerances of marine fungi. Organic carbon concentrations were significantly less throughout the sediments when compared to the site outside of the gyre; the opposite trend was observed with oxygen concentrations. The maximum sediment age sampled was ~76 million years at the outer edge of the gyre.

Long-term isolation within these environments provides a high potential for novel fungal species to adapt unique metabolic strategies for survival, if these populations remain metabolically active. Evolution or adaptation driven by geographic isolation is common with plant and animal populations (Papke and Ward, 2004) and is an important factor in bacterial diversification as a mechanism influencing surface population divergence (Cho and Tiedje, 2000; Papke et al., 2003). However, it is unclear to what extent this extends to single celled eukaryotes such as fungi in deep marine sediments.

The objective of this study was to characterize fungal populations from subsurface SPG sediments collected during Integrated Ocean Drilling Program (IODP) Expedition 329 (D'Hondt et al., 2013). We hypothesized that subsurface fungal populations are metabolically active within isolated SPG sediment with unique metabolisms that aid in their survival. To meet this objective, fungal isolates were cultured from five different sample sites within the gyre and one site at the edge of the gyre at depths ranging from young, shallow sediments to a depth of over 125 mbsf. Morphotypes were determined by microscopy, and growth tolerances and rates were calculated under multiple ecophysiological conditions. RNA was extracted from preserved sediments to construct a clone library to verify *in situ* presence and quantify the *in situ* 18S rRNA gene transcript abundance. Based on genomic taxonomic description (Sobol et al., 2019) and the ecophysiological tests performed herein, we have characterized two unique fungal species.

## Results

### Site Description

**Figure 1:**
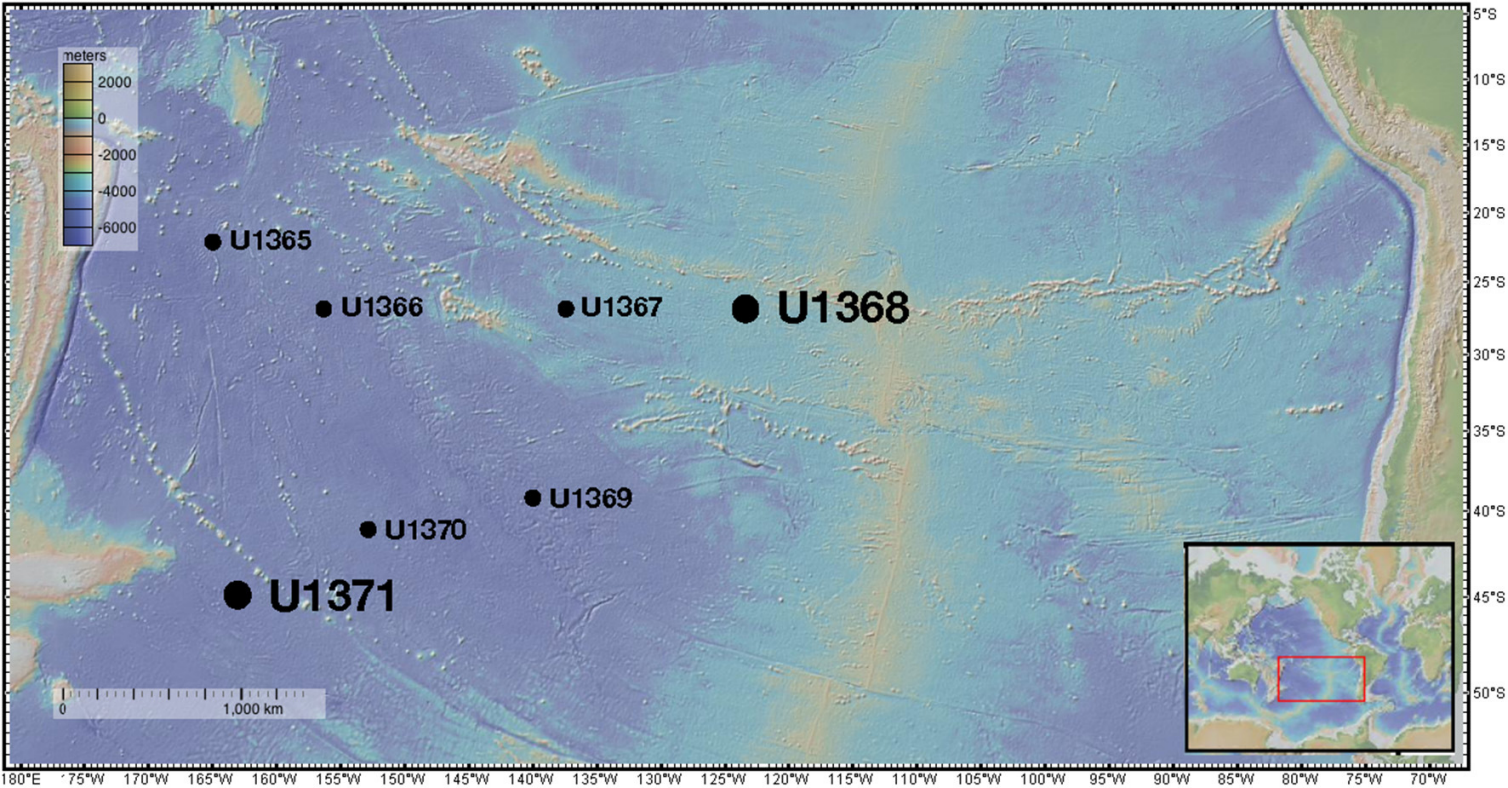
Sampling locations of IODP Expedition 329. The large dots represent the locations from which the two main isolates characterized in this study were derived. Figure was made using GeoMapApp (Ryan et al., 2009).

Sites within the gyre (Figure 1) had low sedimentation rates [0.31–0.89 m million year (myr)^−1^], thin sedimentary cover overlying the basement (<100 m), and low organic carbon burial rates (4.4 × 10 ^−10^-8.2 × 10 ^−9^ mol C/cm^2^ yr^−1^) (Jahnke, 1996; D'Hondt et al., 2009). Prokaryotic cell counts previously reported for these sediments ranged from 10^2^ to 10^6^ cells g^−1^ (D'Hondt et al., 2013). Geochemical analysis indicated that all drill sites within the gyre remained oxic (~100–200 μm O_2_) at the surface to basement (D'Hondt et al., 2013) and dissolved nitrate was present at all depths from ~35 to 45 μm (D'Hondt et al., 2013). In contrast, Site U1371 located outside the gyre, had no detectable oxygen (detection limit ~0.4 μm) below ~5 mbsf (D'Hondt et al., 2013). Dissolved oxygen was not again detected until ~110 mbsf where it was only found to be from 2 μm to 11 μm O_2_ (D'Hondt et al., 2013). Dissolved nitrate is not detected after 2.5 mbsf for site U1371 but returns to ~2–5 μm around ~110 mbsf (D'Hondt et al., 2013). Total organic carbon (TOC) concentrations in U1371 were generally greater than inner gyre sites and ranged from 0.22 wt% at the surface to 0.01 wt% at 128 mbsf (D'Hondt et al., 2013). The geochemical characteristics of U1371 compared to the inner gyre sites suggest that microbial respiration is higher outside the gyre (D'Hondt et al., 2013). The *in situ* temperature in Hole U1371E was 21.2°C at 123.65 mbsf and the pH was 7.69 at 123.65 mbsf. The *in situ* temperature in Hole U1368B was 21.4°C at 12.01 mbsf, and the pH was 7.80 at 12.05 mbsf (Expedition 329 Scientists, 2011). Chloride concentrations were used to estimate salinity. In Hole U1368D at 12.05 mbsf the salinity was ~1.92% and in Hole U137E at 123.65 mbsf it was ~1.99% (Expedition 329 Scientists, 2011). Full geochemical profiles for sediments collected during IODP Expedition 329 were previously reported in D'Hondt et al. (2013).

### Colony Morphology

Isolations from sediment slurry enrichment cultures yielded 18 fungal isolates. Environmental controls maintained in parallel to the sediment samples remained colony free for the entire incubation period. A total of 18 fungal cultures were named SPG-F1 through SPG-F18 and were identified using standard fungal morphological characterization methods (Pitt, 1979). Criteria for characterization included: colony color (obverse and reverse), colony margin, colony texture, hyphae growth, presence of exudates, abundance of conidia, colony structure at 14 days, and rate of conidiogenesis. Morphological characteristics were observed during active growth at 5°C, to match *in situ* temperature, on both the Potato Dextrose Agar (PDA) and Marine Broth Agar (MBA) media types under oxic and anoxic conditions. For all growth conditions, the observed colony characteristics preliminarily grouped the cultures into morphologies that resembled either *Penicillium chrysogenum* or *Penicillium brevicompactum* species.

**Figure 2:**
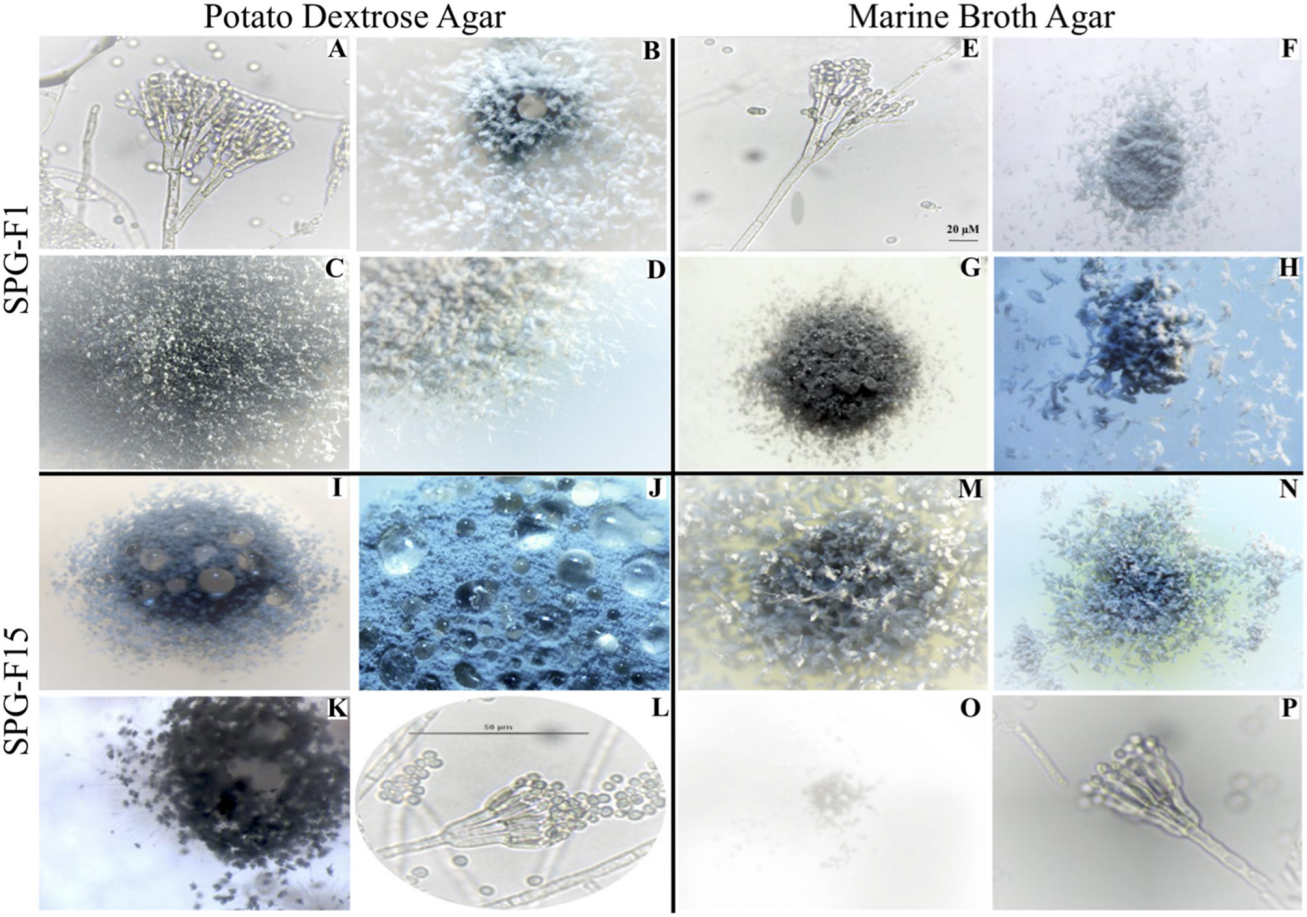
Microscopic images showing morphological characteristics of isolate SPG-F1 from U1371E-14H2 **(A–H)** and isolate SPG-F15 from U1368D-2H1 **(I–P)** grown on either potato dextrose agar **(A–D,I–L)** or marine broth agar **(E–H,M–P)**.

A total of eleven cultures were morphologically homologous to *Penicillium chrysogenum*. These cultures were from samples U1365C-1H2 (SPG-F2), U1365C-9H2 (SPG-F14), U1367D-2H3 (SPG-F11), U1368D-1H2a (SPG-F7), U1368D-1H2b (SPG-F8), U1368D-1H2c (SPG-F8), U1368D-2H4 (SPG-F13), U1369E-1H2a (SPG-F3), U1369E-2H2 (SPG-F12), U1371E-7H2 (SPG-F5), and U1371E-14H2 (SPG-F1) (Table 1). Colony morphology when grown on PDA under oxic conditions at 5°C after 14 days was: 15–20 mm colony diameter, radially sulcate, typically velutinous-flocculent white to yellowish outermost margin, low to moderately deep growth, grayish turquoise to dull green colonies (Munsell colors 24-25D3, 26-27E3-4), yellow-to-yellow brown reverse color, white mycelia, profuse conidiogenesis promptly produced after germination, and most produced pale to light brown exudate with vivid yellow soluble pigment (Figures 2A–D). Colony morphology when grown on PDA under anoxic conditions at 5°C for 14 days was: 13-18 mm colony diameter, radially sulcate, typically velutinous, occasionally centrally floccose, yellow outermost margin, yellow mycelia, delayed low to moderate conidiogenesis, sparse conidiogensis in some, grayish turquoise to dull green colonies (Munsell colors 24-25D3, 26-27E3-4), pale reverse color, most produced exudate, and soluble pigments usually bright yellow. Colony morphology when grown on MBA under aerobic conditions at 5°C for 14 days was: 14–17 mm colony diameter, plain, concentric, dense, umbonate at center, white mycelia, moderate conidiogenesis, grayish to dull green colonies (Munsell colors 26B-E3-4, 27E3-5), pale yellow to pastel yellow reverse color (Munsell color 1A3-4), and absent exudate and soluble pigment. Colony morphology when grown on MBA under anoxic conditions at 5°C for 14 days was: 9–14 mm colony diameter, plain to centrally umbonate, white mycelia, retarded low to moderate conidiogenesis, grayish to dull green colonies (colors: 26B-E3-4, 27E3-5) appearing yellow-green due to the presence of soluble pigment, uncolored to pale reverse color and absent exudate.

**Table 1:** Depth and site location of all isolates from South Pacific Gyre sediments.

**Isolate**	**Site**	**Depth (mbsf)**	**Latitude**	**Longitude**
SPG-F1	U1371E-14H2	124	45°57.8397′S	163°11.0365′W
SPG-F2	U1365C-1H2	2.5	23°51.0377′S	165°38.6502′W
SPG-F3	U1369E-1H2	3	39°18.6070′S	139°48.0246′W
SPG-F4	U1369E-1H2	3	39°18.6070′S	139°48.0246′W
SPG-F5	U1371E-7H2	58.5	45°57.8397′S	163°11.0365′W
SPG-F6	U1368D-1H2	2.6	27°54.9920′S	123°9.6561′W
SPG-F7	U1368D-1H2	2.6	27°54.9920′S	123°9.6561′W
SPG-F8	U1368D-1H2	2.6	27°54.9920′S	123°9.6561′W
SPG-F9	U1369E-2H5	14	39°18.6070′S	139°48.0246′W
SPG-F10	U1369E-2H5	14	39°18.6070′S	139°48.0246′W
SPG-F11	U1367D-2H3	10.7	26°28.8861′S	137°56.3659′W
SPG-F12	U1369E-2H2	9	39°18.6070′S	139°48.0246′W
SPG-F13	U1368D-2H4	13.4	27°54.9920′S	123°9.6561′W
SPG-F14	U1365C-9H2	73	23°51.0377′S	165°38.6502′W
SPG-F15	U1368D-2H1	12	27°54.9920′S	123°9.6561′W
SPG-F16	U1368D-2H1	12	27°54.9920′S	123°9.6561′W
SPG-F17	U1370F-1H3	4.4	41°51.1267′S	139°6.3674′W
SPG-F18	U1370F-1H3	4.4	41°51.1267′S	139°6.3674′W

Isolates from U1368D-2H1a (SPG-F15), U1368D-2H1b (SPG-F16), U1369E-1H2b (SPG-F4), U1369E-2H5a (SPG-F9), U1369E-2H5b (SPG-F10), U1370F-1H3a (SPG-F17), and U1370F-1H3b (SPG-F18) were morphologically homologous to *Penicillium brevicompactum* (Figures 2E–H). Colony morphology when grown on PDA under aerobic conditions at 5°C for 14 days was: 14-17 mm colony diameter, sulcate, velutinous, dense margin, narrow, slightly floccose, white mycelium, moderate conidiogenesis, dull green (Munsell color 25-28D-E3) to dark green (Munsell color 28F5) colonies, yellow reverse, and light brown exudate usually present in minute droplets with no soluble pigment. Colony morphology when grown on PDA under anoxic conditions at 5°C for 14 days was: 10–12 mm colony diameter, velutinous-floccuse, umbonate, white mycelia, delayed low to scarce conidiogenesis, dull green to dark green colonies (Munsell color 27-29E-F4), reverse usually uncolored to pale, and exudate absent or rarely present. Colony morphology when grown on MBA under aerobic conditions at 5°C for 14 days: 10–14 mm colony diameter, plain, usually velutinous, white mycelium, moderate conidiogenesis, usually dull green to very dark green colonies (Munsell color 27-29E-F4), reverse typically yellow and yellow exudate. Colony morphology when grown on MBA under anoxic conditions at 5°C for 14 days: 3-6 mm colony diameter, usually plain, densely constrained, white mycelia, scarce delayed or absent conidiogenesis, dark green colonies (Munsell color 26-27E-G4-7), reverse regularly yellow and yellow exudate.

### Microscopic Characteristics

Based on microscopic identification and colony characteristics, two groups emerged from all isolates that represented either *Penicillium chrysogenum* or *Penicillium brevicompactum*. For more detailed microscopic characteristics, we used the fungal isolate SPG-F1 from U1371E-14H2 as the *Penicillium chrysogenum* representative and isolate SPG-F15 from U1368D-2H1 as the *Penicillium brevicompactum* representative (Figure 2; Supplementary Table 1).

#### Isolate SPG-F1

Conidiophores arose from the agar on stripes and were usually 300–400 μm long, smooth-walled, and hyaline bearing characteristic terverticallite penicillin, with less seldom observations of quaterverticillate (Figures 2A,E). Either one or two mostly terminal and appressed rami were observed to be 13–18 μm long. The philiades were ampuliform and 7–10 μm long and aggregated in clusters of four to seven. The conidia were ellipsoidal to subspheroidal, 2.9–3.5 μm long, smooth-walled and hyaline or slightly greenish (Figure 2A). They were most often observed in long loose columns. The notable difference between growth on Marine Broth or Potato Dextrose was the color of the margins. The colonies of isolate SPG-F1 were green-blue velvety with white-yellow margin (Figures 2B–D,G,H). Reverse color was yellow (Figure 2F).

#### Isolate SPG-F15

Conidiophores were usually borne from shallow and aerial mycelium. Stipes were typically wide, 300–500 μm long, smooth walled and hyaline. The isolate was both terverticillate and quaterverticillate, usually broad, and less often biverticillate (Figures 2L,P). Rami were smooth, usually single and short, 13–16 μm long. Phialides were in verticils, ampuliform, and 6–9 μm long. Conidia were typically ellipsoidal, hyaline, 2.8–3.3 μm long, thick walled, and observed in divergent and disorderly chains. The colonies of SPG-F15 were dark green, dense and constrained with sharp-green margins (Figures 2I–K,M,N). Reverse color was pale (Figure 2O).

### *In situ* Molecular Characterization

**Figure 3:**
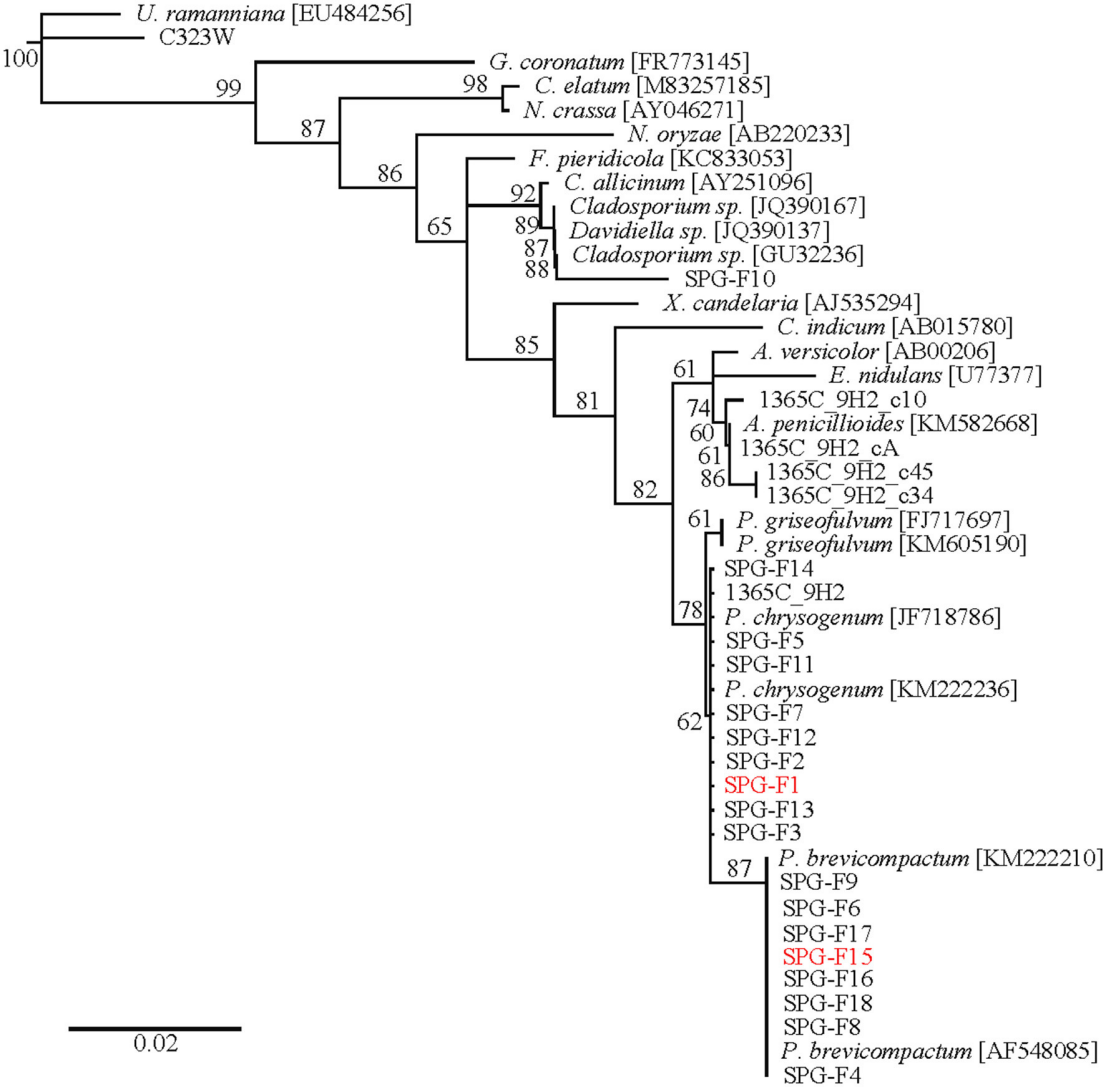
Phylogenetic tree of 18S rRNA gene sequences from clones and isolates from the South Pacific Gyre sediments.

RNA extractions and amplifications from cryopreserved sediment samples were used to construct a clone library. A neighbor-joining phylogenetic tree was created using sequences from a variety of Ascomycetes downloaded from NCBI (Figure 3; Pruitt et al., 2006). The outgroup was *Umbelopsis ramanniana*, which is a member of the outgroup phylum Mucoromycota. Clones U1365C-9H2-c10, -cA, -c45, and -c34 clustered within the Aspergillus clade. Within the *Penicillium chrysogenum* branch were isolates SPG-F1, SPG-F2, SPG-F3, SPG-F5, SPG-F7, SPG-F11, SPG-F12, SPG-F13, and SPG-F14. Placed within the *Penicillium brevicompactum* branch were isolates SPG-F4, SPG-F6, SPG-F8, SPG-F9, SPG-F15, SPG-F16, SPG-F17, and SPG-F18. Isolate SPG-F10 grouped within the *Cladosporium* subclade. Bootstraps >50% supported these phylogenetic placements.

Putatively active *in situ* fungal populations within the sediment from multiple sites and depths were confirmed from quantitative PCR of the 18S rRNA transcripts (Supplementary Figure 1). The sample with the most 18S rRNA transcripts was SPG-F1 (1.8 × 10^4^ per gram sediment) and the least abundant transcripts were in SPG-F13 (7.0 × 10^3^ per gram sediment). The transcript abundance from all analyzed sediment fell within this range.

### Oxygen Tolerance

**Figure 4:**
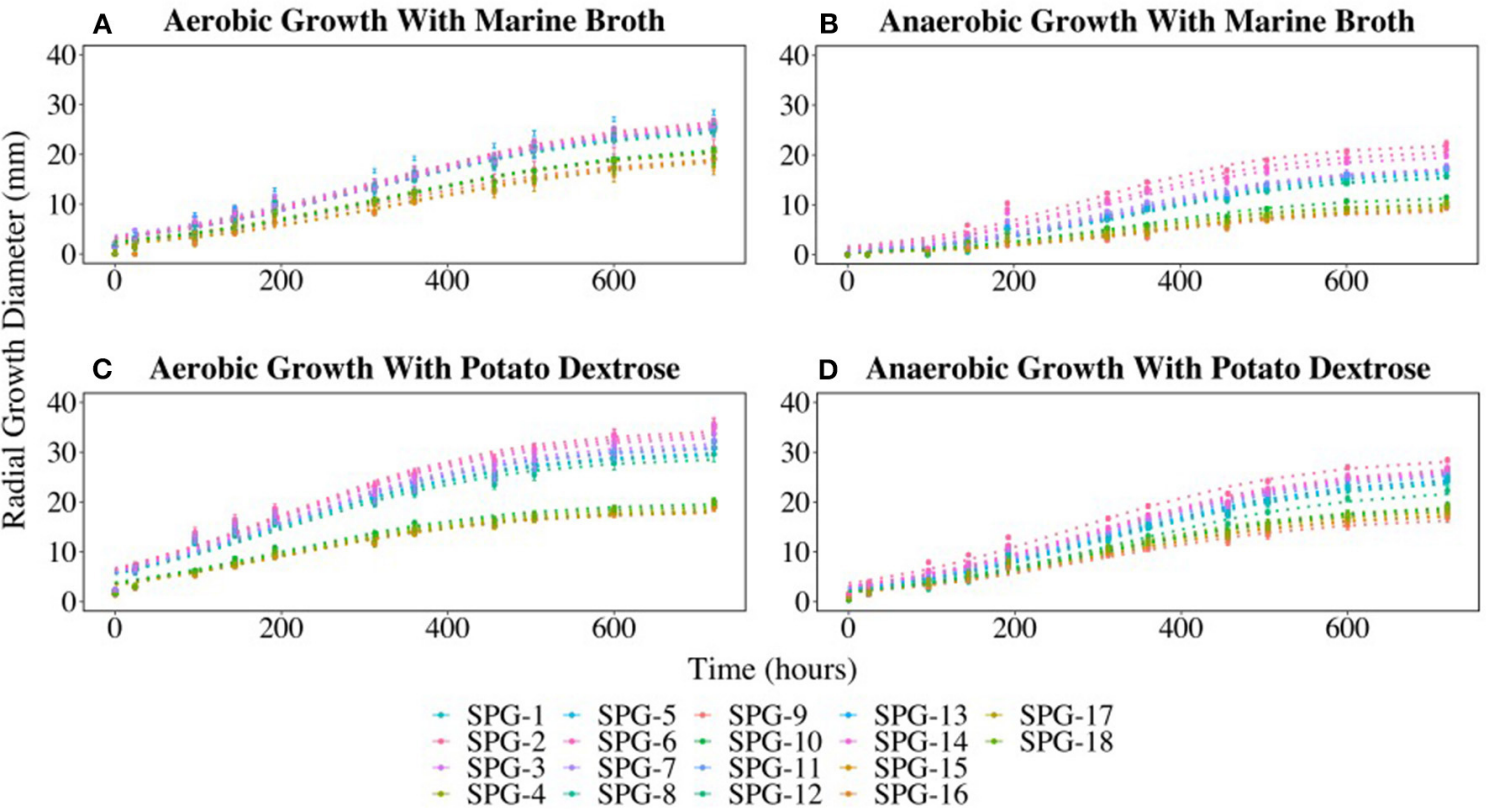
Radial growth rates (in mm) of all isolates on marine broth agar (MBA) or potato dextrose agar (PDA) under oxic or anoxic conditions. **(A)** Aerobic growth with MBA. **(B)** Anaerobic growth with MBA. **(C)** Aerobic growth with PDA. **(D)** Aerobic growth with PDA. Error bars represent the standard error calculated for 12 biological replicates.

All 18 fungal isolates were monitored in triplicate on plates for colony growth rates over a 25-day period. Growth was determined by measuring the diameter of the colony on both the X and Y-planes. All colonies grew on both the marine broth agar (MBA) and potato dextrose agar (PDA) under both oxic and anoxic conditions. Average growth rates were determined and compared for the duration of the growth trial. The 18 fungal isolates grouped into two different growth profiles in three of the four growth conditions tested. The most distinct split between the two groups was observed on MBA in oxic growth conditions where 11 cultures (Group 1) had an average growth rate of 1.2 mm day^−1^, while the remaining 7 cultures (Group 2) had a lower average growth rate of 0.9 mm day^−1^. The growth rates for Group 1 under oxic conditions on MBA were the highest compared to the other three conditions. Similar growth patterns were observed on PDA under anoxic and oxic conditions, with the anaerobic growth rates slightly lower than the aerobic rates (Figure 4).

The growth curves divided into two distinct groups (Figure 4) with Group 1 predominantly initially identified as *P. chrysogenum* based on morphology. Interestingly, two isolates (SPG-F6 and SPG-F8) were morphologically identified as *P. chrysogenum*, but were phylogenetically closely related to *P. brevicompactum* based on initial 18S rRNA gene identification. Group 2 was composed of the remaining strains preliminarily identified as *P. brevicompactum* using morphology, with the exception of SPG-F10 and SPG-F12. The culture from sample SPG-F12 was morphologically and phylogenetically closely related to *P. chrysogenum*. Culture from sample SPG-F10 was morphologically most similar to *P. brevicompactum* but phylogenetically related to *Davidiella* sp, based on the 18S rRNA gene.

The previously observed culture groups diverged into three distinctive subgroups when the cultures were grown on MBA under anoxic conditions (Figure 4). Subgroup 1 was the fastest growing. One of the subgroup 1 strains (SPG-F3) was previously observed in Group 2 when grown in oxic conditions, but had an increased growth rate under anoxic growth conditions (0.7 mm day^−1^). Interestingly, SPG-F4 (preliminarily identified as *P. brevicompactum*) was isolated from the same sediment slurry sample as *P. chrysogenum* SPG-F3, did not show a similar increase in anaerobic growth rate on MBA, but remained in the slower growing subgroup 2. The other two members of the new subgroup 3, SPG-F14 and SPG-F13, were previously in the fast growing Group 1. The remaining members of Group 1 aggregated into the middle of subgroup 3 under these growth conditions.

For the purpose of this study, the two representative isolates were chosen for further analysis to determine the tolerance for temperature, salinity, pH, and lignin degradation, and are described below. Again, SPG-F15 represented the putative genus and cell morphology *P. brevicompactum* from a low organic matter environment and the second isolate, SPG-F1 represented *P. chrysogenum* from a high organic matter environment.

### Temperature Range

**Figure 5:**
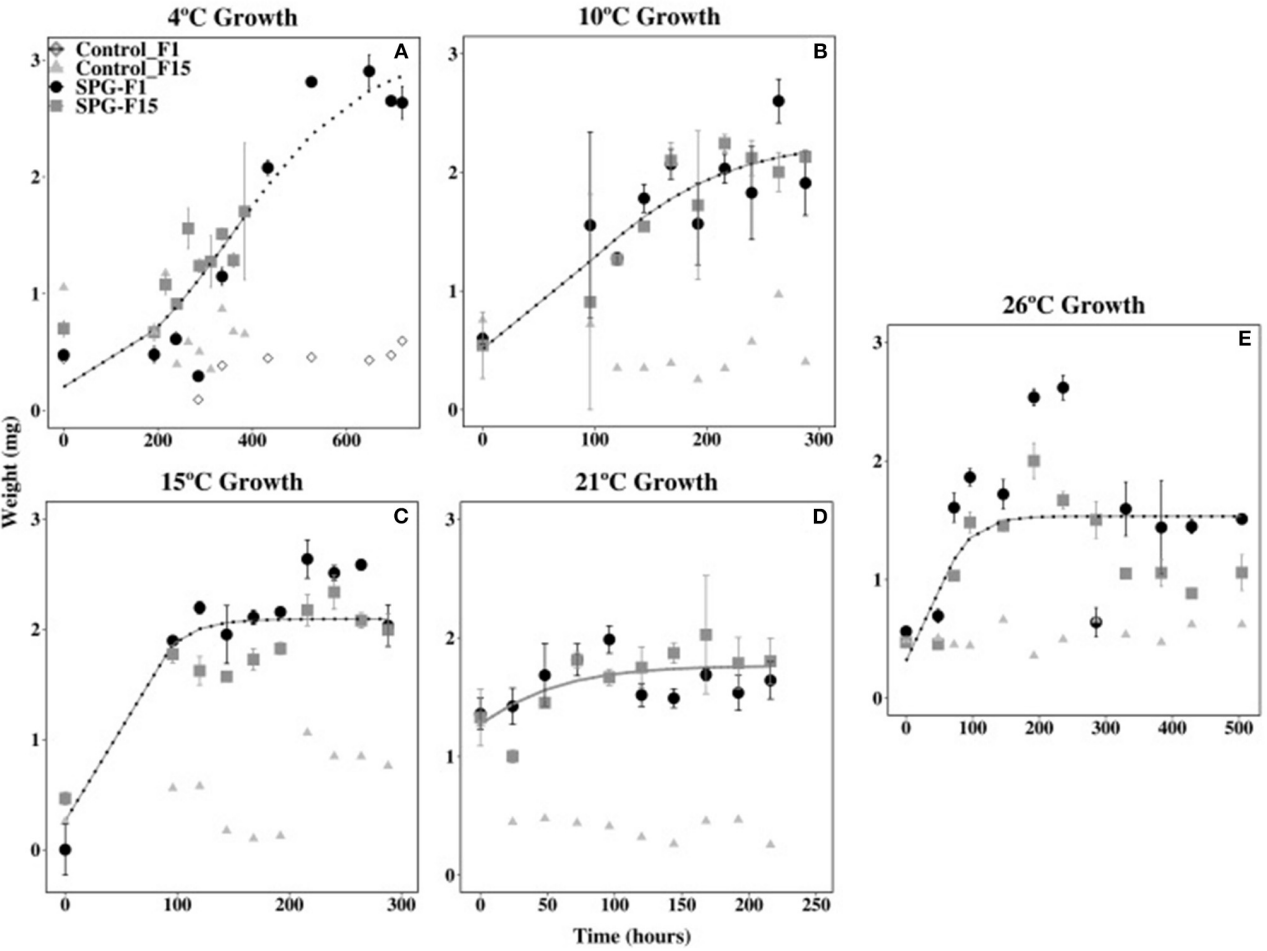
Effect of temperature on SPG-F1 and SPG-F15 growth, measured in mg over time. **(A)** 4°C growth. **(B)** 10°C growth. **(C)** 15°C growth. **(D)** 21°C growth. **(E)** 26°C growth. Only 4°C growth has a control for each isolate because the growth analysis was done at different times. Error bars represent standard error calculated for 3 biological replicates.

Isolates SPG-F1 and SPG-F15 grew at temperatures ranging 4 to 30°C. Growth was not observed at 37°C after 30 days, therefore greater temperatures were not tested. The duration of the lag phase lasted 24 h at 21°C, 48 h at 26°C, 96 h at 10°C and 15°C, and 192 h at 4°C. The exponential growth rates for SPG-F1 were highest (4.39 × 10^−3^ mg h^−1^) at 21°C; whereas, at 4°C, the exponential growth rate was the lowest (1.62 × 10^−3^ mg h^−1^). The highest exponential growth rate for SPG-F15 was also at 21°C (4.81 × 10^−3^ mg h^−1^), but the lowest at 10°C (2.29 × 10^−3^ mg h^−1^). Exponential growth rates were not significantly different (*p* > 0.05) when compared between isolates. This was verified using the predicted Non-linear Mixed-Effects Model (nlme) trends (Figure 5).

The biomass of SPG-F1 steadily decreased after 264 h at 4°C, 216 h at 10°C, 240 h at 15°C, 168 h at 21°C, and 192 h at 26°C. In comparison, isolate SPG-F15 lost biomass after 648 h at 4°C, 264 h at 10°C, 216 h at 15°C, 96 h at 21°C, and 236 h at 26°C. A subsequent increase in biomass was observed after about 200 h in both isolates after the initial decrease. Maximum dry weights did not correlate with optimum growth for both isolates; however, the final dry weight of SPG-F15 was significantly greater than SPG-F1 (*p* = 0.025).

### Salinity Range

Both isolates were able to grow from salinities 0% (same as 21°C growth) to 12% NaCl, but neither grew at 23.4% NaCl. The length of time that both isolates remained in the lag phase of growth increased as NaCl concentrations increased. Both isolates began growing 45.5 h after inoculation at 1 and 2% NaCl, 115 h at 6% NaCl, and 137 h at 8% NaCl. Isolate SPG-F15 grew after 144 h at 12% NaCl, whereas SPG-F1 did not grow until 432 h at the same NaCl concentration (data not shown). The measurements taken at 0 h for both 6% NaCl and 8% NaCl were determined to be outliers in nlme analysis and were therefore removed. This was caused by the leftover salts in the filters and 0 h measurements being heavier than the time at which the isolates began growth.

**Figure 6:**
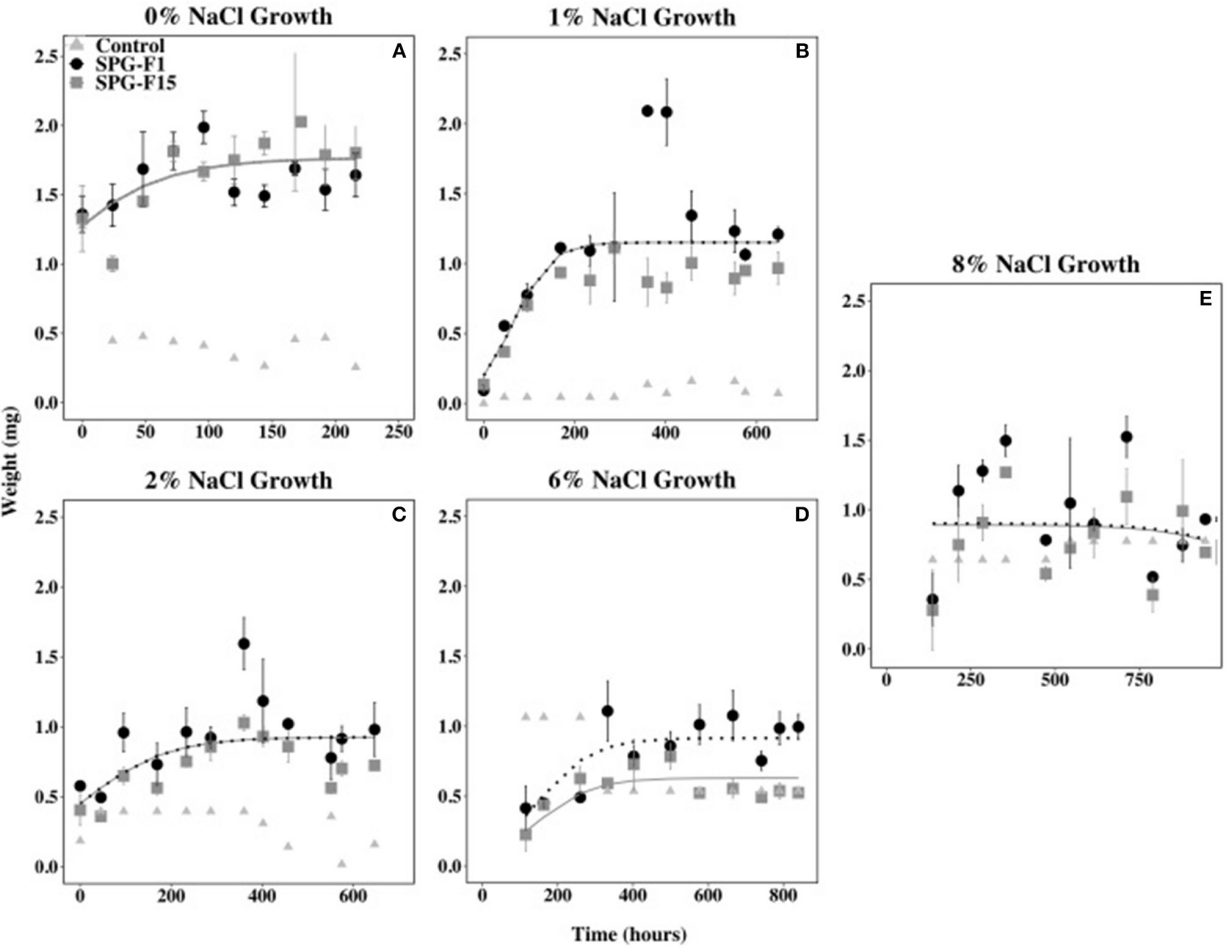
Effect of salinity on SPG-F1 and SPG-F15 growth, measured in mg over time. **(A)** 0% NaCl growth. **(B)** 1% NaCl growth. **(C)** 2% NaCl growth. **(D)** 6% NaCl growth. **(E)** 8% NaCl growth. Time point 0 for 6 and 8% NaCl were removed as they were outliers. Error bars represent standard error calculated for 3 biological replicates.

The growth rate during the exponential phase did not correlate with optimum growth between 0 and 2% NaCl for both isolates. The highest rate during the exponential growth phase for both SPG-F1 and SPG-F15 occurred at 0% NaCl (4.39 × 10^−3^ mg h^−1^ and 4.81 × 10^−3^ mg h^−1^, respectively). The lowest exponential growth rate for SPG-F1 was at 8% NaCl (4.63 × 10^−3^ mg h^−1^); whereas, the lowest exponential growth rate for SPG-F15 was at 2% NaCl (3.00 × 10^−4^ mg h^−1^). According to Student's *T*-test results, there was no significant difference (p>0.05) in growth rates when compared between the fungal isolates. The non-linear mixed-effects (nlme) models predicted no significant difference in growth rates between the isolates, except at 6% NaCl where a clear separation in the trend lines were observed (Figure 6).

The biomass of SPG-F1 steadily decreased after 287 h at 1% NaCl, 360 h at 2% NaCl, 499.5 h at 6% NaCl, and 354 h at 8% NaCl. The biomass for SPG-F15 decreased after 402 h at 1% NaCl, 360 h at 2% NaCl, 332.5 at 6% NaCl, and 354 h at 8% NaCl. No correlation was observed between the time of decrease in biomass and concentration of NaCl. An increase in biomass was observed after the initial decrease in biomass (354 h) for both SPG-F1 and SPG-F15 at 8% NaCl. There was no significant difference between the final maximum dry weights for SPG-F1 and SPG-F15 (*p* = 0.057) according to Student's *T*-test.

### pH Range

We observed growth under acidic (pH 3) to circumneutral (pH 8) conditions in both SPG-F1 and SPG-F15 isolates. Both isolates began growing after 48 h at pH 3, and after 54 h for pH 6. The lag phase lasted 47.5 h for SPG-F15 at pH 8, but SPG-F1 remained in the lag phase for 96 h at pH 8.

**Figure 7:**
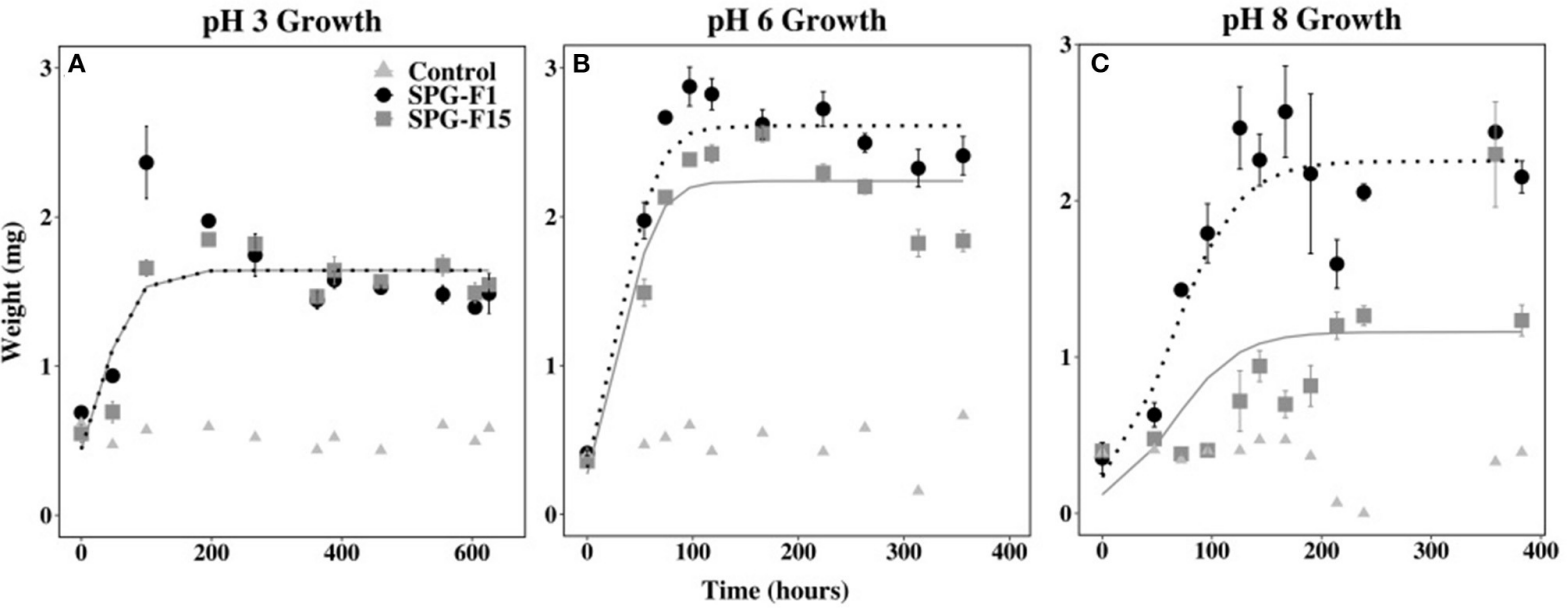
Effect of pH on SPG-F1 and SPG-F15 growth, measured in mg over time. **(A)** pH 3 growth. **(B)** pH 6 growth. **(C)** pH 8 growth. Error bars represent standard error calculated for 3 biological replicates.

The highest exponential growth rates were observed at pH 6 for both SPG-F1 (4.75 × 10^−3^ mg h^−1^) and SPG-F15 (9.32 × 10^−3^ mg h^−1^). The lowest growth rates for both fungi were at pH 8 (SPG-F1 = 2.22 × 10^−3^ mg h^−1^; SPG-F15 = 4.78 × 10^−3^ mg h^−1^). There was no statistically significant difference in growth rates between the isolates at the pH range we tested determined by Student's *T*-test. However, according to nlme models, there was a significant difference in the growth rates of SPG-F1 and SPG-F15 at both pH 6 and pH 8 (Figure 7).

We observed a correlation between the highest growth rates and the time that biomass decreased in both fungi. At pH 6, the biomass for SPG-F1 and SPG-F15 decreased after 97 h and 166 h, respectively. In comparison, the biomass of SPG-F1 decreased after 358.8 and 167 h for SPG-F15 at pH 8. An increase in biomass after the initial decrease was observed at pH 3 for both isolates. Only SPG-F15 increased in biomass at pH 6 and pH 8 after a decline in biomass was observed and the final average maximum biomass weights were significantly greater (*p* = 0.045) for SPG-F15 than the maximum weights for SPG-F1.

### Carbon, Sulfur, and Nitrogen Metabolisms

**Figure 8:**
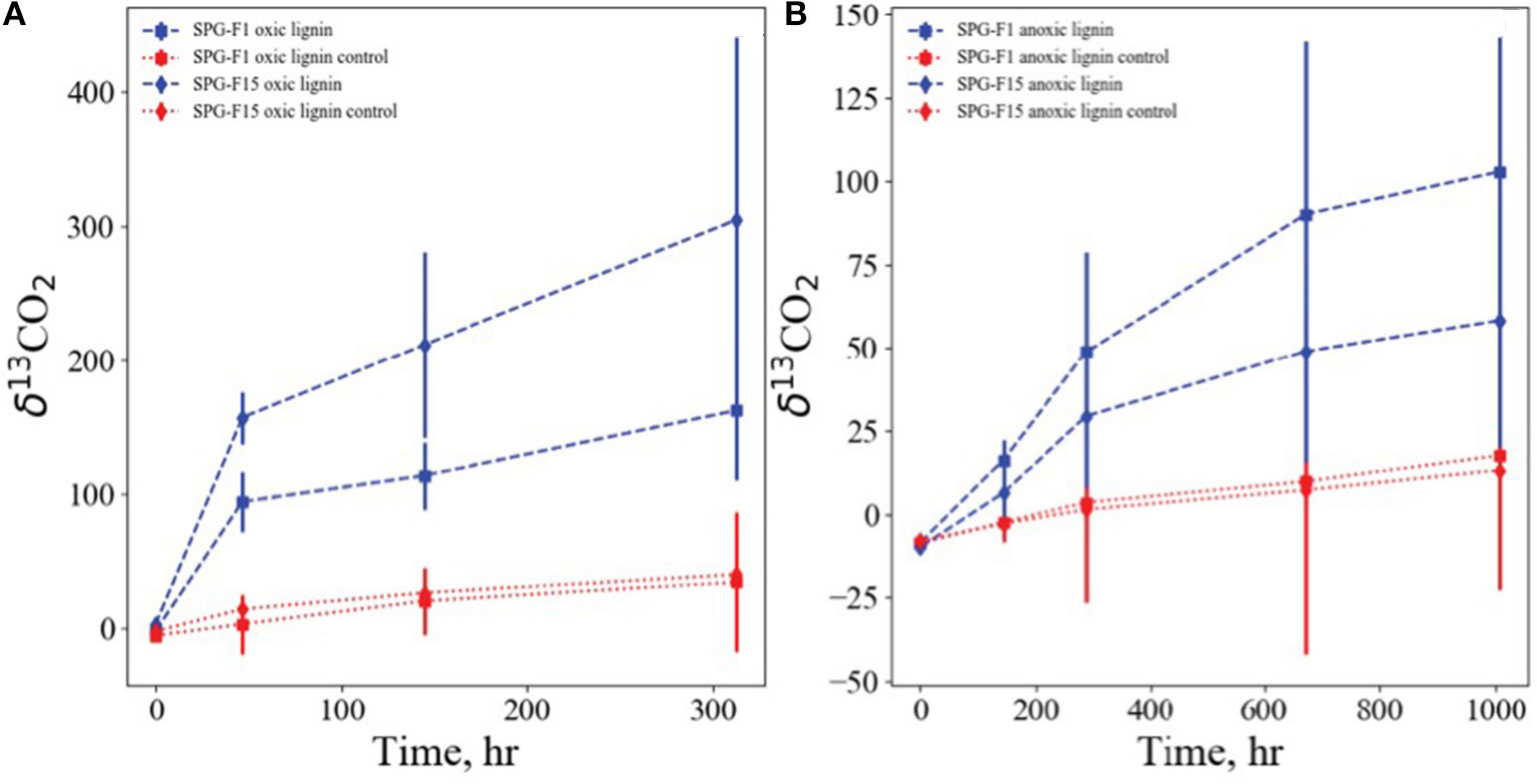
Growth of SPG-F1 and SPG-F15 on isotopically labeled lignin under oxic **(A)** and anoxic **(B)** conditions. Shown are accumulations of lignin derived ^13^C, expressed as δ^13^C-CO_2_ vs. the Vienna PDB standard, in the pool of inorganic carbon over time.

Isolates SPG-F1 and SPG-F15 were incubated with ^13^C labeled lignin, representative of refractory organic matter, under oxic, and anoxic conditions. The fungal biomass was rinsed thoroughly with PBS to remove any residual labile carbon, and no other carbon source was added. Lignin was degraded in oxic and anoxic conditions relative to sterilized control samples. Lignin degradation was faster and more pronounced under oxic conditions with ^13^C derived from labeled lignin accumulating in the inorganic carbon pools of the SPG-F1 and SPG-F15 enrichment, with δ^13^CO_2_ reaching values of 94 and 157 per mil in 48 h, respectively (Figure 8A). In anoxic conditions lignin was also degraded, albeit slower than oxic conditions. For SPG-F1 and SPG-F15, the inorganic carbon pools became enriched in ^13^C, resulting in δ^13^CO_2_ of 48 per mil and 29 per mil in 288 h, respectively (Figure 8B).

Lactose and sucrose was fermented as shown by a change in the media color to yellow (not pictured). Sulfate was found not to be reduced based on both isolates not exhibiting gas production and no black precipitate found in the slants. As for the nitrate reduction tests, gas was not produced and the media remained red after the addition of zinc, therefore both isolates could not reduce nitrate to nitrite or nitrogen gas.

## Discussion

The characterization of metabolically active and viable fungal populations within highly oligotrophic deeply buried marine sediments increases the known limits of eukaryotic life, and at the same time adds another layer of understanding of subsurface carbon cycling. With the acknowledgment of fungi as part of the deep subsurface biosphere, new metabolic processes can be considered when determining global nutrient and carbon cycles. This study advances the understanding of the fungal habitable range by both isolating pure cultures and detecting metabolically active *in situ* populations. Whereas, other studies have taxonomically identified fungi within the deep subsurface (Edgcomb et al., 2011; Orsi et al., 2013; Rédou et al., 2015; Liu et al., 2017), this study is the first from severely resource-limited, deeply buried, sediments with ages up to 70 million years old. IODP Expedition 329 specifically targeted ultra low organic carbon sediments that represent the energetic limits of life (D'Hondt et al., 2013). Fungal hyphae and the conidia (e.g., spores) can be preserved in carbon/nutrient limited and desiccated environments for an undetermined amount of time. A study from Canterbury Basin sediment as deep as 403 mbsf used microscopic visualization to reveal septate fungal filaments, branching fungi, conidiogenesis, and spores indicating preservation, possible dormancy, and potential reanimation of fungi in the subsurface (Pachiadaki et al., 2016). The unique populations that we isolated suggest that the deep subsurface may be a reservoir for novel fungal lineages, potentially new metabolic processes within the eukaryotic domain of life, and preservation of fungi in the deep subsurface.

### Identification and Characterization of SPG Fungi

We are aware that a definitive species identification is not possible with one molecular marker, especially the 18S rRNA gene; however, at the time this was used to simply guide our other experiments and have a general overlook of the fungal community composition. Morphological and genetic characterization of 18 isolates suggested the presence of three different lineages. A majority of the isolates were most closely related to the Ascomycota division. The morphological and genetic identification of each lineage independently supported the lineage identity in all but three isolates. Although the initial phylogenetic analysis using 18S rRNA gene was an important first step to identifying these isolates, we recently determined that isolate SPG-F1 (U1371E-14H2; depth 124 mbsf) and SPG-F15 (U1368D-2H1; depth 12 mbsf) are unique species based on whole genome sequencing of the two fungal isolates (Sobol et al., 2019; Sobol et al. in preparation). Isolate SPG-F1 derived from the location with the greatest organic carbon concentration and isolate SPG-F15 was from the location with the least organic carbon concentration. Their ability to survive and/or thrive under varying ecophysiological conditions was tested herein. The only tests that failed to result in any growth were dissimilatory nitrate/nitrite reduction and dissimilatory sulfate reduction (data not shown). The *in situ* clones were identical to their respective cultured isolates at the same site and depth. This confirms that the cultured isolates were not laboratory contamination and are indigenous to SPG sediments.

All of the isolates appear to be facultative aerobes, yet appear to slightly prefer oxygenic environments based on the overall higher growth rates when exposed to oxygen. The central SPG sedimentary column was oxic throughout where SPG-F15 was isolated from; however, isolate SPG-F1 derived from sediment from just outside the gyre, which became anoxic at 5 mbsf. SPG-F1 grew better aerobically but was from an anoxic environment, which is consistent with fungal physiology. Some fungal species are more tolerant of low oxygen concentrations than others and quite a few terrestrial fungi are facultative anaerobes, including *Penicillium*. It is not uncommon for *Penicillium* to be indifferent to oxygen, switching to homolactic fermentation and even stimulated by high CO_2_ concentrations (Dix, 2012).

In general, fungi are predominantly aerobic, but some members have been observed to be facultative aerobes, relying on fermentation or denitrification (Cathrine and Raghukumar, 2009). Anaerobic fungi have been isolated and/or identified from rumen of herbivores (Haitjema et al., 2014, Kamra and Singh, 2017), gastrointestinal tract (Theodorou et al., 1996), anoxic soils (Kurakov et al., 2008, Tonouchi, 2010), and deep marine sediment (Cathrine and Raghukumar, 2009, Zhang et al., 2014). In order to survive, facultative anaerobic fungi possess specialized organelles called hydrogenosome that use glucose for cellular energy production in the absence of oxygen. The hydrogenase within the hydrogenosome produce molecular hydrogen, carbon dioxide, acetate, and other compounds as metabolic waste products (Ivarsson et al., 2016). Anaerobic fungi are commonly known to produce molecular hydrogen in gut rumen, which contributes to the growth of methanogens. Similarly, fungi have been posited to a source of bioavailable hydrogen in the oceanic crust (Ivarsson et al., 2016), and a source of formate, lactate, acetate forming a consortia with sulfate reducing bacteria in deep granite fractures (Drake et al., 2017).

Isolates SPG-F1 and SPG-F15 were psychrotolerant and mesophilic as demonstrated by growth from 4 to 30°C; however, neither could grow at 37°C. The optimal growth temperature was 21°C. Many fungi can grow over a wide range of temperatures because of their ability to survive in a variety of environmental conditions. *Penicillium* species are notorious for this physiology (Maheshwari et al., 2000, Gunde-Cimerman et al., 2003); which is why they have been found in all types of environments, including marine sediment.

In comparison to the SPG isolates, others found that the closely related species of *P*. *chrysogenum* and *P*. *brevicompactum* from continental environments grew in the range of 5 to 37°C, and optimally at 25 to 30°C (Pitt, 1973; Magan and Lacey, 1984; Sautour et al., 2001; Mahendiran et al., 2013). Additionally, fungi from shallow marine sediments were found to also be mesophilic and psychrotolerant (Singh et al., 2010). However, a *Penicillium* species from brackish mangrove sediments grew optimally at 30°C (Kathiresan and Manivannan, 2006). Interestingly, *P. chrysogenum, P. crustosum*, and *P. canescens* isolates from St. Helena Bay, a relatively cold bay (10 to 14°C), grew from 4 to 37°C, and optimally between 26 and 37°C (Mouton et al., 2012). Although SPG-F1 and SPG-F15 isolates were considered psychrotolerant and mesophilic, they differed from continental and coastal fungi because they did not grow at 37°C.

Previously isolated *P*. *chrysogenum* and *P*. *brevicompactum* species from Canterbury Basin subsurface sediments (12 to 403 mbsf) could grow above 30°C (Rédou et al., 2015), yet these sediments were comparatively much younger and more organic rich. The sediment of Canterbury Basin was estimated to be 4.3 million years old at the terminus (~800 mbsf) (Fulthorpe et al., 2011). TOC concentrations in Canterbury Basin sediments (0.25–1 wt%) were 10 times greater than in SPG sediments. The lower temperature limit of SPG-F1 and SPG-F15, and perhaps losing the ability to grow above 30°C, could be attributed to the differences in *in situ* temperature, sediment age, and TOC concentrations between the two sites. Organic carbon availability has been previously linked to the thermal adaptation of soil fungal communities. In general, studies have shown that low organic carbon concentrations negatively impacted the ability of fungi to grow at higher temperatures (Kirschbaum, 2004, Hartley et al., 2007). The exact physiology behind this finding is uncertain, but another study suggested that differences in fatty acid abundance may distinguish fungi of the same species grown at different temperatures (Stahl and Klug, 1996). Further study is needed about the fatty acids within the fungal isolates from the South Pacific Gyre, and whether fungal fatty acids could have allowed the fungi to grow at greater temperatures.

Although SPG-F1 and SPG-F15 were isolated from sediments with extremely low TOC concentrations (~0.02 wt% at U1371E, <0.002% at U1368D) and at different sediment depths (12 mbsf SPG-F15 vs. 124 mbsf SPG-F1), they both grew at the same rate for all temperatures. This has been observed in one other study that compared three *Penicillium* isolates from continental soils (Abellana et al., 2001), but the reason for such variation was not investigated. It is possible that differences in growth rates were not observed in the current study and in Abellana et al. (2001) because growth rates were determined within the exponential phase of growth. It has been shown that fungi within the same genus have the least amount of variation in growth during the exponential phase (Meletiadis et al., 2001). The transitional periods between exponential growth and stationary growth better determine variation in growth of the same genus (Meletiadis et al., 2001). Future growth rate analysis between similar species isolated from different *in situ* conditions in the marine subsurface may provide more information on the small differences in their growth.

Based on the results of the current study, both SPG-F1 and SPG-F15 isolates were defined as halotolerant because they were capable of growing at 12% NaCl. They also exhibited euryhaline growth because of their ability to grow from 0 to 12% NaCl. However, it was observed that increasing concentrations of NaCl above 2% reduced the growth rates of both isolates. It has been previously found that fungi will reduce growth rates to meet the energy demands the cells experience under salt stress (Albertyn et al., 1994). This adaptive feature could explain why we still saw growth at higher NaCl concentrations, but reduced growth rates. Growth at wide ranges of salinity has also been studied in many fungi, especially *Penicillium* (Tresner and Hayes, 1971; Jones, 2000; Zajc et al., 2012). *Penicillium*, along with most other fungi, have the ability to change the concentrations of glycerol in their membranes to combat osmotic stress brought on by high concentrations of salt (Plemenitaš et al., 2014). The putative adaptation across a wide range of salinities may be another reason why *Penicillium* can be found in a variety of environments such as the marine subsurface.

Neither SPG-F1 nor SPG-F15 grew at hypersaline NaCl concentrations (~24% NaCl). In comparison, one study found that continental *P*. *chrysogenum* could withstand concentrations of 25% NaCl or greater (Tresner and Hayes, 1971). Also, most continental *Penicillium* from the high-altitude soils of the Himalayans grew above 20% NaCl (Dhakar et al., 2014). When we compared these observations to *Penicillium* in marine environments, we found that marine isolates could also grow at NaCl above 20% NaCl. In marine salterns, and the Dead Sea, *P*. *chrysogenum* and *P*. *brevicompactum* both grow to at least 25% NaCl (Zajc et al., 2014). Additionally, *P*. *chrysogenum* from mangrove sediments can grow from 0 to 20% NaCl (Nayak et al., 2012). This evidence suggests that SPG-F1 and SPG-F15 may be physiologically different from continentally derived and coastally derived *Penicillium* because growth was not observed at ~24% NaCl.

The growth of SPG-F1 and SPG-F15 was within range of the *in situ* salinity concentration in SPG sediments, which was ~2%. Similar to this finding, a study of *P*. *chrysogenum* and *P*. *brevicompactum* from Canterbury Basin subsurface sediments found that the isolates also grew best at or near the *in situ* sediment salinity (3% salt concentration) (Rédou et al., 2015). In comparison, *Penicillium* from the coastal marine sediments of St. Helena Bay, had a larger range of optimal growth (1.5–6% salinity) (Mouton et al., 2012). In coastal marine sediments, salinity can change quickly due to large rain events and increases in atmospheric temperature (Atkinson et al., 2007). It may be possible that fungi from coastal sediments have wider salinity optimums in order to survive drastic changes in salinity. However, the marine subsurface is sequestered from such events and the salinity of the sediments does not change drastically. Based on this, it may be possible that marine subsurface fungi are better adapted closer to the *in situ* salinities than coastal sediment fungi (Rédou et al., 2015). The salinity preferences of fungi from other subsurface sediments will help determine if our results are a common occurrence in this environment.

Isolates SPG-F1 and SPG-F15 were classified as acido-tolerant because they could grow from pH 3 to pH 8. Growth at wide ranges of pH has been documented in *Penicillium* before. Continental *Penicillium* have been observed growing between pH 3 and pH 10 (Wheeler et al., 1991). Similarly, in coastal marine sediments of St. Helena Bay, *Penicillium* grew from pH 5 to pH 10. Fungi are notorious for growing across several orders of pH because of the Rim101 pathway that regulates the intake or output of ions into the cell to control internal pH levels (Lamb et al., 2000; Peñalva et al., 2008; Maeda, 2012). It is possible that SPG-F1 and SPG-F15 were capable of growing over a wide range of pH due to this highly conserved pathway, which warrants further genetic analysis.

The optimum growth for SPG-F15 could not be determined between pH 3-8 because, at every pH tested, SPG-F15 began growing ~2 days after inoculation. The same result was observed for SPG-F1, but only between pH 3 and pH 6. Oddly, growth for SPG-F1 at pH 8 did not begin until 4 days after inoculation. Previously reported pH tolerances for continental *Penicillium* species were compared to the pH tolerance of SPG-F1 and SPG-F15. Similarly, in the study of continental *P*. *chrysogenum*, pH did not have a significant impact on germination (Sautour et al., 2001). Similar results have also been reported in coastal marine sediments. In St. Helena Bay sediments, pH did not have a significant impact on the optimal growth for *P. chrysogenum, P. crustosum*, and *P. canescens* between pH 5 to pH 7 (Mouton et al., 2012). Furthermore, *P*. *chrysogenum* isolated from deep sea sediments of the East Pacific did not exhibit significant changes in growth from pH 4 to pH 10 (Luo et al., 2014). Comparing our results to the growth of *Penicillium* in continental environments, coastal sediments, and deep-sea sediments, it appears that pH does not have a significant impact on growth. Again, this may be attributable to the adaptive mechanisms fungi have for tolerating a wide range of pH.

A second growth phase in which the biomass suddenly increased after a steady decline was consistently observed during this study, possibly explained by diauxic shift, the process in which an organism exhausts their preferred energy source and switches to using an alternative source after a lengthy lag-phase (Chu and Barnes, 2016). This shift in growth is commonly observed in the yeast *Saccharomyces cerevisiae*, where cells switch from fermentation of glucose to respiration of ethanol and acetate, indicated by a change in the medium's pH (Smets et al., 2010; Lavaisse et al., 2019). We observed a decrease in pH over time in all growth experiments (data not shown), implying that SPG-F1 and SPG-F15 are too, capable of switching from fermentative to respiratory growth, which could explain the second exponential growth phase we observed.

Another possible explanation behind this increase in growth could be due to the process of autophagy (i.e., “to eat oneself”) (Levine and Klionsky, 2004). During this process, cells respond to stressful conditions such as nutrient starvation by breaking down cytoplasmic components and delivering it to the lysosome to be recycled for nutrients (Levine and Klionsky, 2004). Autophagy related genes (ATG) were found in the genomes of SPG-F1 and SPG-F15 (Sobol et al., 2019). A previous study of *Cryptococcus* and *Aspergillus* isolated from Peru Margin subsurface sediments showed that ATG genes were upregulated compared to continental fungi (Orsi et al., 2015). This increase in the expression of genes related to autophagy is likely due to the limited availability of bioavailable substrates in the Peru Margin sediments (Orsi et al., 2015). The possibility of the fungal isolates in the current study to subsist on autophagy in the nutrient limited sediment of SPG would be highly beneficial and may have played a role in their survival.

Additionally, the delayed-logistic model (Hutchinson, 1948; Forsyth and Caley, 2006) could also explain the oscillating pattern of growth observed for both isolates. According to the model, when a population reaches peak abundance, past their carrying capacity, the population will decrease slightly before increasing again to a more stabilized capacity (Hutchinson, 1948; Forsyth and Caley, 2006). However, further work is needed to determine if visual evidence of hyphae in the SPG sediment cores can be found in order to prove if the fungi are actually growing in the sediments to support the use of autophagy or delayed-logistic growth in this environment. The fluctuation of fungal biomass observed in this study was more likely an artifact of batch culture growth.

### Recalcitrant Organic Matter Degradation

The majority of our understanding of marine fungal metabolism comes from previous studies of continental soil fungi, particularly as it pertains to organic matter degradation. It may be possible that the fungi isolated in this study originated from nearby continents that were deposited via aeolian transport and sedimented over time (Chandralata and Raghukumar, 1998; Spatafora et al., 1998). This has been posited in other marine subsurface environments such as Canterbury Basin sediments (Rédou et al., 2015), deep coal beds off the Shimokita Peninsula in Japan (Liu et al., 2017), and the Central Indian Basin sediments (Damare et al., 2006).

Saprotrophic fungi are key regulators of nutrient and carbon cycles because of their ability to recycle recalcitrant carbon, such as hydrocarbons (Da Silva et al., 2003, Sanyal et al., 2016), lignin (Gubernatorova and Dolgonosov, 2010), and lignocellulose (Riley et al., 2014), into bioavailable substrates and preventing accumulation of dead plant organic matter. Cellulose, hemicellulose, pectin, and lignin comprise the main components of cell walls of plants and lignocellulosic materials. The aromatic rings in the chemical structure of lignocellulose makes it the most recalcitrant component of the plant cell wall and therefore inaccessible to most microorganisms (Cragg et al., 2015). The mechanisms that transform and degrade lignocellulose have been elucidated in most soil white rot fungus, but are still poorly understood in the Ascomycota phyla (Janusz et al., 2017).

Lignin degrading enzymes can be divided into lignin-modifying enzymes (e.g., lignases) and lignin-degrading auxiliary enzymes. A combination of carbohydrate active lignin-modifying enzymes such as phenol oxidase (laccases), heme containing peroxidases, hydrolases, and monooxygenases (Van Dyk and Pletschke, 2012, Cragg et al., 2015) are involved in the degradation of lignocellulose and hemicelluloses in the presence of oxygen. In the current lignin degradation experiment, both isolates showed a statistically significant (*p* < 0.05) increase in growth over the control under oxic conditions by an average of 100% over the total incubation time period. Isolate SPG-F15 grew slightly faster and more abundantly in the oxic conditions, which is consistent with the provenance of the isolate. The sediment from which this isolate came from was located at the center of the South Pacific Gyre and was oxic throughout the sediment column. In contrast, isolate SPG-F1, which came from 124 mbsf in a region outside the gyre in which oxygen was depleted in the top few meters, grew at a higher rate under anoxic conditions. Previous studies have also demonstrated that lignin and lignified plant tissues were biodegradable in the absence of oxygen, although lignocelluloses were recalcitrant to anaerobic biodegradation (Benner et al., 1984). More recently, it was shown that fungi associated with deep-sea sponges (751 m) exhibited strong lignocellulose degrading activity under psychro-, halo-, and pH-tolerant conditions; however, it remains uncertain the precise role the fungi have in this environment (Batista-García et al., 2017). Nonetheless, it is clear that marine-derived fungi appear to be a good source for novel and important metabolites for biotechnological applications.

Within the genomes of both SPG fungi described here, there was evidence of carbohydrate active enzymes (CAZy) capable of lignin degradation (Sobol et al., 2019). Lignin degradation has been previously identified in continental soil fungi, namely *P. chrysogenum* (Rodriguez et al., 1994). Within the CAZy group of enzymes, the most abundant auxiliary activities (AA) families in both isolates, AA3 (glucose-methanol-choline oxidoreductase), AA7 (glucooligosaccharide oxidase), GH3 (β-Glucosidase/β-xylosidase), and GH5 (endoglucanase) have been documented in wood-decaying fungi for the oxidation of carbohydrates in lignocellulose (Levasseur et al., 2013, Riley et al., 2014, Takahashi et al., 2015, Nagy et al., 2016, Schneider et al., 2016). The paucity of organic carbon in SPG sediments (<0.01%) and the relatively high abundance of inorganic carbon, much of which appears to have been derived from terrestrial input made up of plant matter (D'Hondt et al., 2009; Estes et al., 2019), lead us to surmise that fungal populations like the ones we isolated in this study rely on their ability to degrade recalcitrant organic matter for survival. Both SPG isolates are potentially important members of the carbon cycle if they metabolize and transform the recalcitrant organic matter in SPG sediments; therefore, future studies of carbon cycling in the marine subsurface should take into account the carbon cycling activity by fungi.

## Conclusion

We successfully cultured fungi from oligotrophic sediment within SPG that were ~11–70 million years old and confirmed them to also be metabolically active in the sediment. Isolates SPG-F1 and SPG-F15 were capable of surviving in a range of temperatures, salinity, and pH but grew best at or near *in situ* SPG sediment conditions. Both isolates could not grow at 37°C nor at 25% NaCl, suggesting that they may be physiologically different from continentally derived and coastal marine sediment *Penicillium*. Overall, this study sheds light on the ecophysiology of two *Penicillium* isolates from SPG sediments and expands the currently known boundaries for eukaryotic life.

## Materials and Methods

### Sample Collection and Processing

During the Integrated Ocean Drilling Program (IODP) Expedition 329, sediment was collected from six drill sites along a transect into and back out of center of the South Pacific Gyre (U1365, U1367, U1368, U1369, U1370, and U1371) and sectioned following standard IODP core flow (Expedition 329 Scientists, 2011). Sites U1365 and U1371 were located on the outer edge of the gyre, whereas sites U1367-U1370 were located within the gyre along two transects (Figure 1). From core material collected at these sites, eleven core sections were selected for analysis representing varying depths and proximity to the center of the gyre. All interior core sections used in this study for subsampling passed standard shipboard perfluorocarbon tracer (PFT) analysis indicating core integrity was maintained during coring and core recovery, and no drill fluid or seawater contaminated the collected sediments (Expedition 329 Scientists, 2011). Sediment cores were retrieved in 9.5-m core liners that were cut into 1.5-m sections. These sections were immediately sub-sectioned into 5-cm whole round core sections and stored at 4°C for culture-based analysis and −80°C for molecular-based analysis. Sample subsections are named using the following convention: U1365C-1H2; U1365 is the drill site, C is the third replicate hole at the drill site, 1 is the first 9.5 m core retrieved, H indicates hydraulic piston core, and 2 is the second 1.5-m section cut from the top of the 9.5-m core. Subsections for the cultured-based analysis included U1365C-1H2 [2.3 m below seafloor (mbsf)], U1365C-9H2 (72.7 mbsf), U1367D-2H3 (10.5 mbsf), U1368D-1H2 (2.3 mbsf), U1368D-2H4 (12.0 mbsf), U1369E-1H2 (2.5 mbsf), U1369E-2H2 (9.3 mbsf), U1369-2H5 (13.3 mbsf), U1370F-1H3 (3.1 mbsf), U1371E-7H2 (58.3 mbsf), and U1371E-14H2 (124.2 mbsf). Sediment for culturing was shipped chilled and stored at 4°C and whole round cored designated for *in situ* molecular analyses was stored and shipped at −80°C.

### Enrichment Cultures

Sediment slurries were constructed in 125 ml glass septum bottles using 30 g of sediment stored at 4°C and 100 ml of Difco 2216 marine broth (BD Diagnostics; Franklin Lakes, NJ, USA). Control bottles were constructed in parallel with only marine broth added and no sediment. All bottles were incubated at 5°C in the dark and shaking periodically at 100 rpm for 1 year. Following incubation for 1 year, 100 μl of media from the sediment slurries and the control bottles were sterilely extracted through the septum and plated on marine broth agar (1.5% agar) plates and incubated at 5°C in the dark. After 1 month of growth on the plates, fungal strains were subcultured onto new marine broth agar and potato dextrose agar (Difco 2134) plates. Fungal cultures were maintained by subculturing every 3 months onto fresh media plates. Long-term preservation was achieved by cryopreservation of fungal plugs in 10% glycerol and DMSO and stored at −80°C. During every manipulation of the cultures, un-inoculated control plates were exposed to all environmental conditions. All control plates and sediment slurries remained sterile throughout the experimental period.

### Molecular Analysis

RNA was extracted from frozen (−80°C) whole round cores using the method described in Reese et al. (2013). Control extractions using DNA/RNA-free water in place of sediment were extracted simultaneously to confirm lack of contamination during the extraction method. The 18S ribosomal RNA (rRNA) were reverse transcribed for 30 min at 37°C using MMLV reverse transcriptase and the universal fungal 18S rRNA primer nu-SSU-1196 to produce complementary DNA (cDNA). Fungal gene transcripts were quantified from the cDNA following Lloyd et al. (2010). We used an Applied Biosystems Step-One Plus (Life Technologies, Grand Island, NY, USA) with the Quantitect SYBR Green PCR kit (Qiagen, Foster City, CA, USA) according to manufacturer's recommended conditions followed by a melt curve analysis for verification of single product amplification. Primers nu-SSU-0817 and nu-SSU-1196 (Borneman and Hartin, 2000) were used with the following program: 94°C for 5 min followed by 40 cycles of 94°C for 30 s, 56°C for 30 s, and 72°C for 30 s, with a final extension at 72°C for 10 min. Standards were prepared from amplified 18S rRNA fungal genes purified by gel elution (Qiaquick Gel Elution; Qiagen, Foster City, CA, USA) and quantified using a Nanodrop ND-1000 spectrophotometer (ThermoFisher, Wilmington, DE, USA). The 18S rRNA fungal standards were diluted over five orders of magnitude and amplified with four replicates at each dilution (*r*^2^ = 0.996). Sensitivity was calculated with precision to 10^1^ target molecules per reaction. The standard curve was used to quantify the unknown samples. All controls were performed as customary for qPCR (Orcutt et al., 2013).

An aliquot of the 18S rRNA gene PCR product, produced using the same primers and program as described for qPCR, was gel purified (Qiaquick Gel Elution; Qiagen, Foster City, CA, USA) and cloned using the TOPO-TA 2.1 Kit (Invitrogen, Life Technologies, Grand Island, NY, USA). Positive transformants were picked for plasmid miniprep using the PureLink Quick Plasmid Miniprep Kit (Invitrogen, Life Technologies, Grand Island, NY, USA). Purified minipreps (500 ng) were amplified with T3 and T7 primers (5 pmol ml^−1^) following the manufacturer's instructions, and sent to Retrogen sequencing facility (San Diego, CA, USA) for Sanger sequencing. Sequences were deposited in NCBI GenBank under accession number SUB9765307.

Sequences of the 18S rRNA gene from all SPG fungal isolates and retrieved sequences from GenBank (Benson et al., 2017) were aligned using Geneious version 8.1.9 (Kearse et al., 2012). The alignment type used was a global alignment with free end gaps. The alignment was manually trimmed to 326 bp in Geneious. Phylogenetic relationships were determined using the Jukes-Cantor genetic distance model and the Neighbor-joining tree building method. The support for each node was determined by 1,000 replications of bootstrap analysis.

### Morphological Characterization of Cultures

Potato dextrose agar (PDA) and marine broth agar (MBA) plus streptomycin (0.02 mg mL-1) were used for fungal morphological characterization. Each plate was inoculated three times with a single fungal strain in an equilateral triangle pattern using a sterile needle. Each fungal strain was cultured in duplicate on both media types. To assess the ability for facultative anaerobic growth anaerobically prepared PDA and MBA media were similarly inoculated with each fungal strain in an anoxic chamber, sealed, and placed in BD GasPak EZ pouch systems with an oxygen indicator to ensure anoxic conditions. Aerobic and anaerobic inoculated plates were incubated in the dark at 5°C. Control plates not containing fungi but exposed to the same manipulation were also incubated under the same conditions.

Fungal growth characteristics and morphology were determined for both isolates in each growth condition. Growth rates were calculated from the average of two perpendicular diameters of the colony taken every 24–48 h until the colony achieved maximum growth. Colony texture and macroscopic characteristics were examined under a Nikon SMZ800 dissecting microscope (Feasterville, PA, USA). Obverse and reverse color was recorded under artificial light illumination and designated according to Munsell Color Charts (Munsell and Munsell, 1929).

High-resolution microscopic examination of fungal structure was produced on wet mount slides using a Nikon Ci-E at 400× magnification. Conidiophore structure including length, phialides, branching system and conidia were recorded. Characterization and species descriptions followed methods previously described (Pitt, 1973; Toru Okuda et al., 2000; Pitt and Hocking, 2009).

For the purpose of this study, two representative isolates were chosen from the site location with the greatest organic carbon concentration (U1371) and the least concentration (U1368). The isolate from U1371E-14H2 (45°57.8397′S, 163°11.0365′W) was collected at 124 mbsf and was given the name SPG-F1. The isolate from U1368D-2H1 (27°54.9920′S, 123°9.6561′W) was located at a depth of 12 mbsf and was given the name SPG-F15.

### Spore Isolation

A spore isolation method was modified using a combination of previously published protocols (Ho and Ko, 1997; Choi et al., 1999; Zhang et al., 2013). In brief, pure fungal cultures were grown on 20% potato dextrose agar (Becton Dickson; Franklin Lakes, NJ, USA) plates supplemented with streptomycin (Thermo Fisher; Waltham, MA, USA) to a final concentration of 0.05 mg ml^−1^. The fungi were incubated for 1 week at 30°C for optimum spore production. After sporulation, 5 mL of 0.2% SDS was pipetted directly onto the plate help remove the spores. The SDS and spore suspension was filtered through 315 μm mesh (General Oceanics; Miami, FL, USA) and centrifuged at room temperature at 8,000 rpm for 3 min. The spores were counted using a hemocytometer under a standard light microscope (Olympus; Tokyo, Japan).

### Physiological Characterization

We measured the growth rates of SPG-F1 and SPG-F15 at various temperatures (4, 10, 15, 21, 26, 30, 37, 45, and 60°C), salinities (1, 2, 4, 6, 8, and 23.4% NaCl), pH (2, 3, 4, 6, 8, and 10), and oxygen tolerance. An aliquot of spores (1 x 10^4^ final concentration) was inoculated in 10 mL of potato dextrose broth that was diluted to 20% of the manufacturer's recommendation. The media was supplemented with streptomycin at a final concentration of 0.05 mg mL^−1^. Adding NaCl to the potato dextrose broth altered salinity. A minimum growth period of 10 days and a maximum of 30 days was used for each growth test. Three biological replicates were included for each time point and triplicate potato dextrose broth media blanks (i.e., no spores added) were included for each test to assess contamination.

Growth was determined by measuring the dry weight of the fungal biomass using a modified protocol (Taniwaki et al., 2006). At the predetermined time points, each of the culture bottles were filtered onto pre-dried and pre-weighed GF/F filters (24 mm diameter, 1.1 μm porosity). The pre-dried filters were dried at 80°C for 18 h and upon cooling, the initial weight was recorded to a precision of 0.001 mg. The fungal biomass was gently filtered and dried at 80°C for 18 h. The final dry weight for each replicate was determined by subtracting the pre-weight from the post-weight. The pH of the media was measured prior to filtration using a SevenCompact pH meter (Mettler Toledo; Hong Kong, China). Due to salt interferences with the salinity tests, the filters were rinsed twice with 5 mL of sterile water prior to filtration.

The ability to reduce nitrate via dissimilatory denitrification was analyzed using the Greiss Reagent method (Knapp and Clark, 1984). A 1-mL aliquot of culture was inoculated in 25 × 150-mm test tubes that contained 25 mL of nitrate broth (pH 7.2) and Durham tubes were used to test for gas production. The ability to reduce sulfate, and ferment glucose, lactose, and/or sucrose was tested using Triple Sugar Iron (TSI) agar slants (Hajna, 1945).

### Incubations With ^13^C-Labeled Carbon Sources

We investigated the capacity of isolates SPG-F1 and SPG-F15 to breakdown lignin under oxic and anoxic conditions. To do this, we aseptically grew a large stock of fungal biomass aerobically in nutrient-rich marine broth with antibiotic streptomycin (final concentration 0.05 mg mL^−1^). The fungal cultures were poured into 50-mL centrifuge tubes and centrifuged (<500 rpm). The nutrient-rich media comprising the supernatant was discarded and the fungi were rinsed with phosphate buffering solution (PBS) a total of three times. Using a wide-bore pipette tip, we removed ~100 mL of concentrated biomass and placed it in a headspace vial containing 10 mL of artificial porewater appropriate for the treatment. The artificial porewater consisted of the following: 413 mM NaCl, 53 mM ${\text{MgCl}}_{2}^{*}$6H_2_O, 28 mM Na_2_SO_4_, 11 mM KCl, 10 mM ${\text{CaCl}}_{2}^{*}$2H_2_O, 2.6 mM NaHCO_3_, 0.03 mM KNO_3_, 0.01 mM NH_4_Cl, and 0.001 mM KH_2_PO_4_. The artificial porewater was autoclaved and purged with N_2_ as it cooled for anoxic treatment or simply cooled for oxic treatments, and streptomycin was added. After biomass was added, anoxic treatment vials were immediately capped with thick butyl rubber stoppers and purged with N_2_ to remove oxygen. A thin butyl septa was placed on the oxic treatments. In order to discriminate the breakdown of specific substrates, we amended headspace vials containing fungal sample with treatments of ^13^C-lignin (3.2 mg mL^−1^, 100 mL) in triplicate. The substrates were purged with N_2_ gas prior to addition to the incubations. Immediately after addition of substrate, the initial time-point was taken. This consisted of taking a headspace sample with a gas tight syringe and measuring the δ^13^C–CO_2_ via GC-IRMS (Thermo Fisher, Bremen, Germany). All time points after were carried out in the same manner. Controls with no template included and using only the oxic or anoxic media plus the labeled substrate were carried throughout the experiment.

### Data Analysis

Average growth rates, measured as dry weight (milligrams) over time (hours), were determined during the exponential growth phase. Growth rates were calculated as the natural log from the difference in weights during the exponential phase, over time (Equation 1). N_0_ represents the dry weight of the fungi at the beginning of the exponential phase, N_t_ represents the dry weight at the end of the exponential phase, Δ_t_ represents the change in time, and rate of change (Andersen, 2005).


(1) $$\begin{array}{r} r=\frac{\ln\left(\text{Nt}-\text{N}0\right)}{\Delta \text{t}} \end{array}$$

Student's *T*-test were performed using Microsoft Excel® Analysis Tool-Pak to determine significant differences in growth rates using a *p*-value cutoff of 0.05. We created non-linear mixed-effect models (nlme) to model the fungal growth trends over time using the nlme function in the nlme library in R (Version 1.1.383) (Pinheiro et al., 2017).

## Data Availability Statement

The datasets presented in this study can be found in online repositories. The names of the repository/repositories and accession number(s) can be found in the article/Supplementary Material.

## Ethics Statement

Ethical review and approval or specific consent procedures were not required for this study in accordance with the local legislation and institutional requirements.

## Author Contributions

BK and MS conceived the manuscript, performed experiments, analyzed data, and wrote the manuscript. MB and K-UH assisted in experimentation, analyzed data, and edited the manuscript. All authors contributed to the article and approved the submitted version.

## Conflict of Interest

The authors declare that the research was conducted in the absence of any commercial or financial relationships that could be construed as a potential conflict of interest.

## Publisher's Note

All claims expressed in this article are solely those of the authors and do not necessarily represent those of their affiliated organizations, or those of the publisher, the editors and the reviewers. Any product that may be evaluated in this article, or claim that may be made by its manufacturer, is not guaranteed or endorsed by the publisher.

## Supplementary Material

Supplementary Figure 1 18S rRNA transcript abundance within the sediment from SPG sites.

Supplementary Table 1 Colony characteristics.

Supplementary Table 2 Microscopic characteristics.

Supplementary Table 3 Growth rate at each treatment. Growth rate at 0% salinity and at 21^o^C were determined at the same time.
