# Supplementary figures and images for: Redefining the Subsurface Biosphere: Characterization of Fungi Isolated From Energy-Limited Marine Deep Subsurface Sediment
Source: Front Fungal Biol. 2021 Sep 24;2:727543. doi: 10.3389/ffunb.2021.727543 (PMC10512353; doi:10.3389/ffunb.2021.727543)

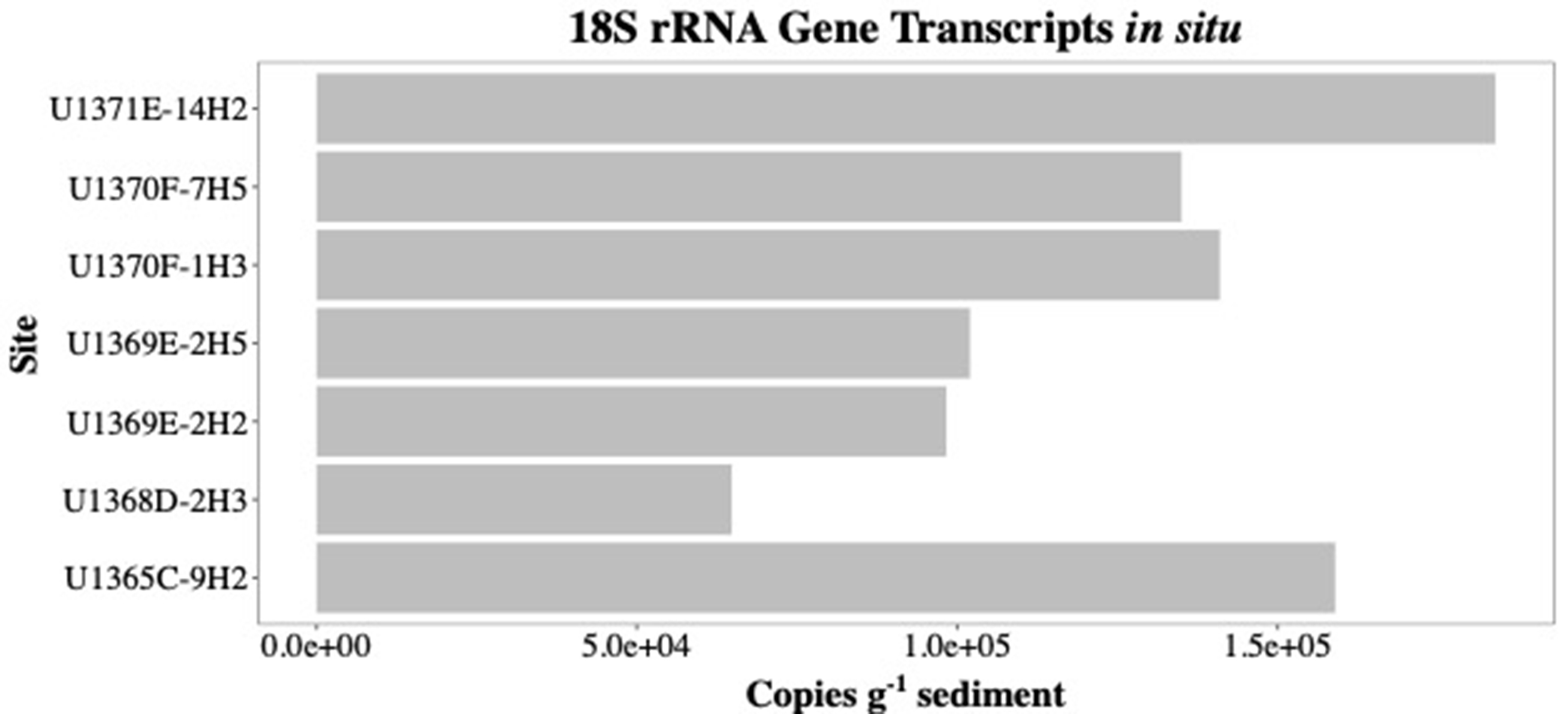

Supplement: Supplementary Figure 1 — 18S rRNA transcript abundance within the sediment from SPG sites. [file Image_1.jpg]
