# Supplementary material for: Redefining the Subsurface Biosphere: Characterization of Fungi Isolated From Energy-Limited Marine Deep Subsurface Sediment
Source: Front Fungal Biol. 2021 Sep 24;2:727543. doi: 10.3389/ffunb.2021.727543 (PMC10512353; doi:10.3389/ffunb.2021.727543)
Supplement: Supplementary Table 1 — Colony characteristics. [file Data_Sheet_1.pdf]

Growth Condition	Isolate	Colony Color	Margin Hyphae	Color Reverse	Colony Texture	Hyphae	Exudate	Conidia	Colony at 7 days	Conidiogenesis
Aerobic PDA 5 °C	SPG-F1	Green-blue	White yellow	yellow	Velutinous-Floccuse	Submerged	Light brown	Profuse	Microcolony	promptly
	SPG-F2	Green-blue	White yellow	yellow	Velutinous-Floccuse	Submerged	Light brown	Profuse	Microcolony	promptly
	SPG-F3	Green-blue	White yellow	yellow	Velutinous-Floccuse	Submerged	Light brown	Profuse	Microcolony	promptly
	SPG-F4	Dark green	Green	light yellow	Dense-constrained	Submerged	Brown abundant	Moderate	Microcolony	promptly
	SPG-F5	Green-blue	White yellow	yellow	Velutinous-Floccuse	Submerged	Light brown	Profuse	Microcolony	promptly
	SPG-F6	Green-blue	White yellow	yellow	Velutinous-Floccuse	Submerged	Light brown	Profuse	Microcolony	promptly
	SPG-F7	Green-blue	White yellow	yellow	Velutinous-Floccuse	Submerged	Light brown	Profuse	Microcolony	promptly
	SPG-F8	Green-blue	White yellow	yellow	Velutinous-Floccuse	Submerged	Light brown	Profuse	Microcolony	promptly
	SPG-F9	Dark green	Green	light yellow	Dense-constrained	Submerged	Brown abundant	Moderate	Microcolony	promptly
	SPG-F10	Dark green	Green	light yellow	Dense-constrained	Submerged	Brown abundant	Moderate	Microcolony	promptly
	SPG-F11	Green-blue	White yellow	yellow	Velutinous-Floccuse	Submerged	Light brown	Profuse	Microcolony	promptly
	SPG-F12	Green-blue	White yellow	yellow	Velutinous-Floccuse	Submerged	Light brown	Profuse	Microcolony	promptly
	SPG-F13	Green-blue	White yellow	yellow	Velutinous-Floccuse	Submerged	Light brown	Profuse	Microcolony	promptly
	SPG-F14	Green-blue	White yellow	redish yellow	Velutinous-Floccuse	Submerged	Light brown	Profuse	Microcolony	promptly
	SPG-F15	Dark green	Green	pale	Dense-constrained	Submerged	Brown abundant	Moderate	Microcolony	promptly
	SPG-F16	Dark green	Green	pale	Dense-constrained	Submerged	Brown abundant	Moderate	Microcolony	promptly
	SPG-F17	Dark green	Green	light yellow	Dense-constrained	Submerged	Brown abundant	Moderate	Microcolony	promptly
	SPG-F18	Dark green	Green	light yellow	Dense-constrained	Submerged	Brown abundant	Moderate	Microcolony	promptly

Growth Condition	Isolate	Colony Color	Margin Hyphae	Color Reverse	Colony Texture	Hyphae	Exudate	Conidia	Colony at 7 days	Conidiogenesis
Anaerobic PDA 5 °C	SPG-F1	Green-blue	White yellow narrow	pale	Velutinous-Floccuse	Submerged	Light brown	Moderate	Microcolony	retarded
	SPG-F2	Green-blue	White yellow narrow	pale	Velutinous-Floccuse	Submerged	Light brown	Moderate	Microcolony	retarded
	SPG-F3	Green-blue	White yellow narrow	pale	Velutinous-Floccuse	Submerged	Light brown	Moderate	Microcolony	retarded
	SPG-F4	White-center green	White	white	Elevated-sulcate-constrained	Submerged	Brown abundant	Scarse	Microcolony	retarded
	SPG-F5	Green-blue	White yellow narrow	pale	Velutinous-Floccuse	Submerged	Light brown	Moderate	Microcolony	retarded
	SPG-F6	Green-blue	White yellow narrow	pale	Velutinous-Floccuse	Submerged	Light brown	Moderate	Microcolony	retarded
	SPG-F7	Green-blue	White yellow narrow	pale	Velutinous-Floccuse	Submerged	Light brown	Moderate	Microcolony	retarded
	SPG-F8	Green-blue	White yellow narrow	pale	Velutinous-Floccuse	Submerged	Light brown	Moderate	Microcolony	retarded
	SPG-F9	White	White	white	Elevated-sulcate-constrained	Submerged	Brown abundant	Scarse	Microcolony	retarded
	SPG-F10	White	White	white	Elevated-sulcate-constrained	Submerged	Brown abundant	Scarse	Microcolony	retarded
	SPG-F11	Green-blue	White yellow narrow	pale	Velutinous-Floccuse	Submerged	Light brown	Moderate	Microcolony	retarded
	SPG-F12	Green-blue	White yellow narrow	pale	Velutinous-Floccuse	Submerged	Light brown	Moderate	Microcolony	retarded
	SPG-F13	Green-blue	White yellow narrow	pale	Velutinous-Floccuse	Submerged	Light brown	Moderate	Microcolony	retarded
	SPG-F14	Green-blue	White yellow narrow	pale	Velutinous-Floccuse	Submerged	Light brown	Moderate	Microcolony	retarded
	SPG-F15	White	White	white	Elevated-sulcate-constrained	Submerged	Brown abundant	Scarse	Microcolony	retarded
	SPG-F16	White-center green	White	white	Elevated-sulcate-constrained	Submerged	Brown abundant	Scarse	Microcolony	retarded
	SPG-F17	White	White	white	Elevated-sulcate-constrained	Submerged	Brown abundant	Scarse	Microcolony	retarded
	SPG-F18	White	White	white	Elevated-sulcate-constrained	Submerged	Brown abundant	Scarse	Microcolony	retarded

Growth Condition	Isolate	Colony Color	Margin Hyphae	Color Reverse	Colony Texture	Hyphae	Exudate	Conidia	Colony at 7 days	Conidiogenesis
Aerobic MB 5 °C	SPG-F1	Grey-green	hyaline	pale	Plane - concentric	Submerged	absent	Moderate	Microcolony	promptly
	SPG-F2	Grey-green	hyaline	pale	Plane - concentric	Submerged	absent	Moderate	Microcolony	promptly
	SPG-F3	Grey-green	hyaline	pale	Plane - concentric	Submerged	absent	Moderate	Microcolony	promptly
	SPG-F4	Dark green	yellow	yellow	Dense-constrained	Submerged	yellow	Moderate	Microcolony	promptly
	SPG-F5	Grey-green	hyaline	pale	Plane - concentric	Submerged	absent	Moderate	Microcolony	promptly
	SPG-F6	Grey-green	hyaline	pale	Plane - concentric	Submerged	absent	Moderate	Microcolony	promptly
	SPG-F7	Grey-green	hyaline	pale	Plane - concentric	Submerged	absent	Moderate	Microcolony	promptly
	SPG-F8	Grey-green	hyaline	pale	Plane - concentric	Submerged	absent	Moderate	Microcolony	promptly
	SPG-F9	Dark green	yellow	yellow	Dense-constrained	Submerged	yellow	Moderate	Microcolony	promptly
	SPG-F10	Dark green	yellow	yellow	Dense-constrained	Submerged	yellow	Moderate	Microcolony	promptly
	SPG-F11	Grey-green	hyaline	pale	Plane - concentric	Submerged	absent	Moderate	Microcolony	promptly
	SPG-F12	Grey-green	hyaline	pale	Plane - concentric	Submerged	absent	Moderate	Microcolony	promptly
	SPG-F13	Grey-green	hyaline	pale	Plane - concentric	Submerged	absent	Moderate	Microcolony	promptly
	SPG-F14	Grey-green	hyaline	pale	Plane - concentric	Submerged	absent	Moderate	Microcolony	promptly
	SPG-F15	Dark green	yellow	yellow	Dense-constrained	Submerged	yellow	Moderate	Microcolony	promptly
	SPG-F16	Dark green	yellow	yellow	Dense-constrained	Submerged	yellow	Moderate	Microcolony	promptly
	SPG-F17	Dark green	yellow	yellow	Dense-constrained	Submerged	yellow	Moderate	Microcolony	promptly
	SPG-F18	Dark green	yellow	yellow	Dense-constrained	Submerged	yellow	Moderate	Microcolony	promptly

Growth Condition	Isolate	Colony Color	Margin Hyphae	Color Reverse	Colony Texture	Hyphae	Exudate	Conidia	Colony at 7 days	Conidiogenesis
Anerobic MB 5 °C	SPG-F1	Hyaline-green center	hyaline	pale	Plane - concentric	Submerged	absent	Moderate	Microcolony	retarded
	SPG-F2	Hyaline-green center	hyaline	pale	Plane - concentric	Submerged	absent	Moderate	Microcolony	retarded
	SPG-F3	Hyaline-green center	hyaline	pale	Plane - concentric	Submerged	absent	Moderate	Microcolony	retarded
	SPG-F4	Green-yellowish	yellow	yellow	Constrained	Submerged	yellow	scarse	Microcolony	retarded
	SPG-F5	Hyaline-green center	hyaline	pale	Plane - concentric	Submerged	absent	Moderate	Microcolony	retarded
	SPG-F6	Hyaline-green center	hyaline	pale	Plane - concentric	Submerged	absent	Moderate	Microcolony	retarded
	SPG-F7	Hyaline-green center	hyaline	pale	Plane - concentric	Submerged	absent	Moderate	Microcolony	retarded
	SPG-F8	Hyaline-green center	hyaline	pale	Plane - concentric	Submerged	absent	Moderate	Microcolony	retarded
	SPG-F9	Green-yellowish	yellow	yellow	Constrained	Submerged	yellow	scarse	Microcolony	retarded
	SPG-F10	Green-yellowish	yellow	yellow	Constrained	Submerged	yellow	scarse	Microcolony	retarded
	SPG-F11	Hyaline-green center	hyaline	pale	Plane - concentric	Submerged	absent	Moderate	Microcolony	retarded
	SPG-F12	Hyaline-green center	hyaline	pale	Plane - concentric	Submerged	absent	Moderate	Microcolony	retarded
	SPG-F13	Hyaline-green center	hyaline	pale	Plane - concentric	Submerged	absent	Moderate	Microcolony	retarded
	SPG-F14	Hyaline-green center	hyaline	pale	Plane - concentric	Submerged	absent	Moderate	Microcolony	retarded
	SPG-F15	Green-yellowish	yellow	yellow	Constrained	Submerged	yellow	scarse	Microcolony	retarded
	SPG-F16	Green-yellowish	yellow	yellow	Constrained	Submerged	yellow	scarse	Microcolony	retarded
	SPG-F17	Green-yellowish	yellow	yellow	Constrained	Submerged	yellow	scarse	Microcolony	retarded
	SPG-F18	Green-yellowish	yellow	yellow	Constrained	Submerged	yellow	scarse	Microcolony	retarded
