# Supplementary material for: Redefining the Subsurface Biosphere: Characterization of Fungi Isolated From Energy-Limited Marine Deep Subsurface Sediment
Source: Front Fungal Biol. 2021 Sep 24;2:727543. doi: 10.3389/ffunb.2021.727543 (PMC10512353; doi:10.3389/ffunb.2021.727543)
Supplement: Supplementary Table 2 — Microscopic characteristics. [file Data_Sheet_2.pdf]

Growth Condition	Isolate	Penicilli	Philiades	Conidia	Stipes Length (µM)	Rami #	Rami length (µM)	Philiades Length (µM)	Conidia Length (µM)
Aerobic PDA 5 °C	SPG-F1	terverticalli	ampulliform	subspheroidal	289.7	2	17.2	9.3	3.0
	SPG-F2	quaterverticalli	ampulliform	subspheroidal	280.9	2	17.5	8.8	3.3
	SPG-F3	quaterverticalli	ampulliform	subspheroidal	281.3	2	17.8	8.8	3.2
	SPG-F4	quaterverticalli	ampulliform	ellipsoidal	526.9	1	14.6	6.9	3.1
	SPG-F5	quaterverticalli	ampulliform	subspheroidal	283.6	2	17.3	8.9	3.0
	SPG-F6	quaterverticalli	ampulliform	subspheroidal	286.9	2	16.7	9.0	3.2
	SPG-F7	quaterverticalli	ampulliform	subspheroidal	286.3	2	17.4	9.2	3.3
	SPG-F8	quaterverticalli	ampulliform	subspheroidal	286.3	2	17.3	9.3	3.2
	SPG-F9	terverticalli	ampulliform	ellipsoidal	514.7	1	14.8	7.1	2.9
	SPG-F10	terverticalli	ampulliform	ellipsoidal	534.3	1	14.0	7.3	3.0
	SPG-F11	quaterverticalli	ampulliform	subspheroidal	285.0	2	17.5	9.3	3.3
	SPG-F12	quaterverticalli	ampulliform	subspheroidal	284.5	2	17.5	9.3	3.4
	SPG-F13	quaterverticalli	ampulliform	subspheroidal	286.4	2	17.3	9.0	3.1
	SPG-F14	quaterverticalli	ampulliform	subspheroidal	283.9	2	17.0	8.9	3.3
	SPG-F15	terverticalli	ampulliform	ellipsoidal	524.8	1	14.6	6.9	3.0
	SPG-F16	terverticalli	ampulliform	ellipsoidal	526.0	1	13.8	7.0	3.1
	SPG-F17	terverticalli	ampulliform	ellipsoidal	531.7	1	14.4	7.7	3.1
	SPG-F18	terverticalli	ampulliform	ellipsoidal	527.6	1	14.4	6.7	3.1
Growth Condition	Isolate	Penicilli	Philiades	Conidia	Stipes Length (µM)	Rami #	Rami length (µM)	Philiades Length (µM)	Conidia Length (µM)
Anaerobic PDA 5 °C	SPG-F1	terverticalli	ampulliform	subspheroidal	277.0	2	17.8	9.0	3.2
	SPG-F2	quaterverticalli	ampulliform	subspheroidal	277.7	2	17.4	9.4	3.0
	SPG-F3	quaterverticalli	ampulliform	subspheroidal	277.0	2	17.4	8.5	3.2
	SPG-F4	quaterverticalli	ampulliform	ellipsoidal	511.7	1	14.3	6.9	3.1
	SPG-F5	quaterverticalli	ampulliform	subspheroidal	277.3	2	18.4	9.2	3.1
	SPG-F6	quaterverticalli	ampulliform	subspheroidal	277.4	2	18.0	9.7	3.3
	SPG-F7	quaterverticalli	ampulliform	subspheroidal	277.3	2	17.6	8.8	3.2
	SPG-F8	quaterverticalli	ampulliform	subspheroidal	278.5	2	18.3	9.0	3.3
	SPG-F9	terverticalli	ampulliform	ellipsoidal	511.3	1	13.8	7.5	3.1
	SPG-F10	terverticalli	ampulliform	ellipsoidal	511.3	1	13.4	6.9	2.9
	SPG-F11	quaterverticalli	ampulliform	subspheroidal	277.1	2	18.6	9.2	3.3
	SPG-F12	quaterverticalli	ampulliform	subspheroidal	277.5	2	17.9	9.1	3.2
	SPG-F13	quaterverticalli	ampulliform	subspheroidal	276.8	2	17.6	9.3	3.0
	SPG-F14	quaterverticalli	ampulliform	subspheroidal	277.0	2	18.2	9.3	3.2
	SPG-F15	terverticalli	ampulliform	ellipsoidal	508.2	1	13.8	7.3	3.0
	SPG-F16	terverticalli	ampulliform	ellipsoidal	507.6	1	14.1	6.8	3.0
	SPG-F17	terverticalli	ampulliform	ellipsoidal	512.4	1	13.4	7.0	3.0
	SPG-F18	terverticalli	ampulliform	ellipsoidal	508.5	1	13.4	7.2	3.1
