# Supplementary material for: Redefining the Subsurface Biosphere: Characterization of Fungi Isolated From Energy-Limited Marine Deep Subsurface Sediment
Source: Front Fungal Biol. 2021 Sep 24;2:727543. doi: 10.3389/ffunb.2021.727543 (PMC10512353; doi:10.3389/ffunb.2021.727543)
Supplement: Supplementary Table 3 — Growth rate at each treatment. Growth rate at 0% salinity and at 21oC were determined at the same time. [file Data_Sheet_3.pdf]

		Growth Rate (μg/hr)	
		**SPG-F1**	**SPG-F15**
**Temperature**	**4ºC**	1.619	2.742
	**10ºC**	2.647	2.293
	**15ºC**	2.608	4.494
	**21ºC**	4.390	4.813
	**26ºC**	3.043	4.272
**Salinity**	**0%**	4.390	4.813
	**1%**	0.106	1.925
	**2%**	1.273	0.303
	**4%**	0.683	1.116
	**6%**	1.508	2.104
	**8%**	0.046	0.604
**pH**	**3**	2.445	5.188
	**6**	4.750	9.322
	**8**	2.217	4.775
